# Regulation of PDF receptor signaling controlling daily locomotor rhythms in *Drosophila*

**DOI:** 10.1371/journal.pgen.1010013

**Published:** 2022-05-23

**Authors:** Weihua Li, Jennifer S. Trigg, Paul H. Taghert

**Affiliations:** Department of Neuroscience, Washington University School of Medicine, St Louis, Missouri, United States of America; Universidad de Valparaiso, CHILE

## Abstract

Each day and in conjunction with ambient daylight conditions, neuropeptide PDF regulates the phase and amplitude of locomotor activity rhythms in *Drosophila* through its receptor, PDFR, a Family B G protein-coupled receptor (GPCR). We studied the *in vivo* process by which PDFR signaling turns off, by converting as many as half of the 28 potential sites of phosphorylation in its C terminal tail to a non-phosphorylatable residue (alanine). We report that many such sites are conserved evolutionarily, and their conversion creates a specific behavioral syndrome opposite to loss-of-function phenotypes previously described for *pdfr*. That syndrome includes increases in the amplitudes of both Morning and Evening behavioral peaks, as well as multi-hour delays of the Evening phase. The precise behavioral effects were dependent on day-length, and most effects mapped to conversion of only a few, specific serine residues near the very end of the protein and specific to its A isoform. Behavioral phase delays of the Evening activity under entraining conditions predicted the phase of activity cycles under constant darkness. The behavioral phenotypes produced by the most severe PDFR variant were ligand-dependent *in vivo*, and not a consequence of changes to their pharmacological properties, nor of changes in their surface expression, as measured *in vitro*. The mechanisms underlying termination of PDFR signaling are complex, subject to regulation that is modified by season, and central to a better understanding of the peptidergic modulation of behavior.

## Author summary

In multi-cellular organisms, circadian pacemakers create output as a series of phase markers across the 24 hour day to allow other cells to pattern diverse aspects of daily rhythmic physiology and behavior. Within circadian pacemaker circuits, neuropeptide signaling is essential to help promote coherent circadian outputs. In the fruit fly *Drosophila* 150 neurons are dedicated circadian clocks and they all tell the same time. In spite of such strong synchronization, they provide diverse phasic outputs in the form of their discrete, asynchronous neuronal activity patterns. Neuropeptide signaling breaks the clock-generated symmetry and drives many pacemakers away from their preferred activity period in the morning. Each day, neuropeptide PDF is released by Morning pacemakers and delays the phase of activity of specific other pacemakers to later parts of the day or night. When and how the PDF that is released in the morning stops acting is unknown. Furthermore, timing of signal termination is not fixed because day length changes each day, hence the modulatory delay exerted by PDF must itself be regulated. Here we test a canonical model of G protein-coupled receptor physiology to ask how PDF receptor signaling is normally de-activated. We use behavioral measures to define sequence elements of the receptor whose post-translational modifications (e.g., phosphorylation) may define the duration of receptor signaling.

## Introduction

In *Drosophila*, neuropeptide PDF signaling helps pattern the output of the fly circadian pacemaker network that controls rhythmic daily locomotor activity [1–3]. Its functions have been compared to those of neuropeptide VIP in regulating mammalian circadian physiology [4]. Historically PDF was first isolated as an active principle (Pigment Dispersing Hormone) that mediates light-dependent dispersion of pigment granules in diverse chromatophores of crustacea [5,6]. In the insect circadian system, PDF acts for a specified period within each 24 hr cycle, and works in conjunction with environmental light to set phase and amplitude for locomotor activity rhythms that normally occur around dawn and dusk [7–9]. Each day, the precise times of dusk and of dawn change, which alters the time interval between them. These facts require that the time of PDF signaling, the point when it starts and the point when it stops, must also be adjusted each day to appropriately follow and reflect these daily variations in the light: dark transitions. PDF signaling starts following its release by specific pacemaker neurons, whose period of activity *in vivo* tracks the dawn in a variety of photoperiodic conditions [10]. We lack a comparable understanding of how PDF receptor signaling normally stops: this work addresses that mechanism.

The PDF receptor (PDFR) is a member of the Family B (secretin receptor-like) GPCR group [11–13]: it is G_s_-coupled and its activation elevates cAMP levels *in vivo* [14]. It regulates different adenylate cyclases (AC) in diverse target pacemakers [15,16], which, through PKA activation, ultimately regulate the pace of the molecular clock through regulation of Timeless [17]. PDFR autoreceptor signaling promotes dramatic, daily morphological changes in the axonal terminals of sLNv pacemakers [18]. In addition to its effects on the pace of the molecular pacemaker, PDFR activation also regulates calcium dynamics in subsets of pacemaker neuron groups to help dictate their group-specific, daily phases of activation (in the sLNv, in the 5^th^ sLNv and in subsets of LNd, and DN3 groups [10]). Such target cell-specific delays of PER-dependent neuronal activity illustrate the basis by which the circadian network produces a daily series of staggered phasic, neuronal outputs [19,20]. Finally, PDF/PDFR signaling is long-lasting: its depression of basal calcium levels in target neurons persists without abatement over many hours [19]. These observations raise fundamental questions regarding the mechanism and the time course by which PDFR signaling diminishes in anticipation of the next day’s cycle of signaling.

The canonical model of GPCR phosphorylation and homologous desensitization features G protein-coupled receptor kinases (GRKs) which associate with activated GPCRs and phosphorylate cytosolic segments, thereby recruiting β-arrestins [21,22]. β-arrestins uncouple the receptors from G proteins [23,24]. or enhance receptor endocytosis [25]; they can also serve as signal transducers by recruiting distinct signaling molecules [26]. A second major regulatory mechanism to reduce GPCR signaling is heterologous desensitization, whereby second-messenger-dependent kinases (PKA or PKC) phosphorylate GPCRs [27]. Thus, we designed experiments to modify evolutionarily-conserved residues in the C terminal tail of PDFR that could conceivably serve as substrates for phosphorylation and subsequent signal termination. Our working hypothesis was that, by their actions *in vivo*, such modified PDF receptors would reveal extended lifetimes of activation.

We know very little about the mechanisms that underlie normal termination of PDFR signaling. In the small LNv, *pdfr* levels are higher late in the day than in early morning [28]. Sensitivity to PDF *in vivo* peaks in the early day and is regulated by the PER-dependent clock, via post-transcriptional mechanisms: the EC50 for PDF responses in identified neurons varies systematically 5–10 fold: as a consequence of RalA action, as a function of time of day, and as a function of seasonality [29]. PDFR signaling is long-lasting: it persists for many hours *in vivo* [19], for as long as free peptide ligand is available in the bath [14]. In addition, β-arrestin2-GFP is not efficiently recruited to activated PDFR when the receptor is functionally-expressed in *hEK-293T* cells [29]. In contrast, each of 13 other *Drosophila* neuropeptide GPCRs (including two Family B GPCRs, CG8422 and CG17415) efficiently recruit β-arrestin2-GFP, when they are activated by their cognate ligands in that cellular environment [30–32].

Here we report (i) that PDFR is normally phosphorylated *in vivo* at conserved C terminal residues; (ii) that loss of conserved PDFR phosphorylatable sites leads to a behavioral syndrome opposite to loss-of-function *pdf* and *pdfr* phenotypes, with effects on both the amplitude and phase of the daily locomotor peaks. This ‘gain of function’ approach reveals a multi-hour range of potential phases for both the Morning and Evening activity peaks, within which neuropeptide PDF:PDFR signaling normally specifies rhythmic behavior, according to season. In addition, using a structure-function approach, this work identifies specific PDFR sequence elements that are major points at which the duration of receptor activity is regulated, and through which behavior is modulated in season-specific fashion.

## Results

### PDFR C-Terminal sequences

**Fig 1:**
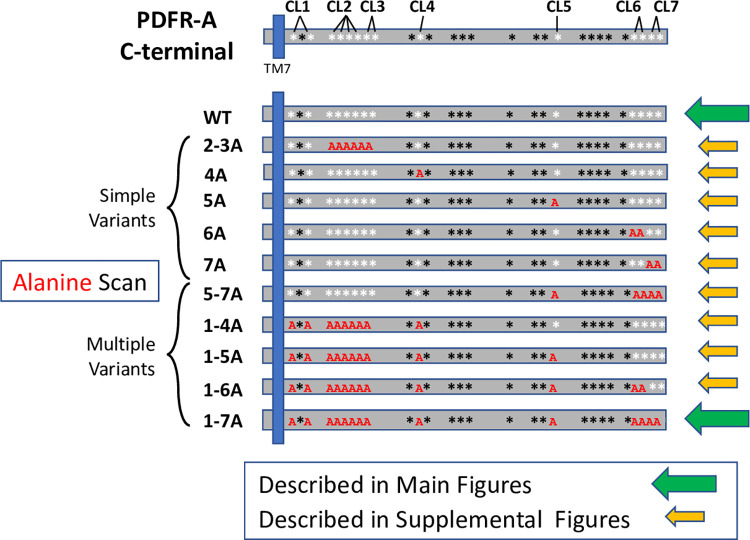
A map illustrating the series of PDFR sequence variants used to test a role for PDFR phosphorylation in regulating the locomotor rhythms. The C Terminal of the PDFR-A isoform is diagrammed (top) to the right of the 7^th^ transmembrane domain (TM7, blue). Asterisks indicate the positions of the 28 serine, threonine or tyrosine residues present within the PDFR C Terminal tail. The residues marked by the white asterisks were those chosen for study by sequence alteration to encode Alanine; they were given arbitrary designations as Clusters (CL) 1 through 7. Each cluster contains a single residue (e.g., CL4 or CL5), or as many as four residues (e.g., CL2). The ten sequence variants are named by the Cluster(s) that was altered followed by the letter ‘A”. Simple Variants include those wherein residues in one or two Clusters were altered. Multiple Variants include those wherein residues within three or more Clusters were altered. All 14 targeted residues were altered in the PDFR 1-7A variant. The green arrows mark the WT and 1-7A PDFR variant: their behavioral effects are featured in the Main Figures. The yellow arrows mark the nine other PDFR variants: their behavioral effects are described in **S2 Fig, S3 Fig, S4 Fig, S5 Fig, S6 Fig** and **S7 Fig**.

Based on alternative splicing, the *pdfr* locus in *Drosophila melanogaster* (CG13758) encodes very similar GPCRs which differ in their extreme C terminal sequences, for which the PA and PD isoforms are representative (flybase.org/reports/FBgn0260753). The PD isoform is slightly longer and lacks the final ~20 AAs of the PA isoform. We focused on the PA protein isoform of PDFR, as it has been used in the majority of genetic studies in the field (**S1 Fig**). To identify residues for mutation, we first used comparative genomic analyses to assess how well specific sequences in the C-terminal region of the PA receptor are conserved. We obtained annotated *pdfr* genomic sequences from 16 additional species of *Drosophila*, in both the *Sophophora* and *Drosophila* sub-families (**S1 Table**). Together this species collection represents an estimated 40–60 MYr of *Drosophalid* evolution [33]. We defined residue V505 of the *melanogaster* protein as the start of the C terminal sequence, following the consensus 7^th^ transmembrane TM domain (TM7) (**S1 Fig**). These 17 different PDFRs all contain C-termini of considerable length, and vary between 189 to 215 amino acids (AAs). The *melanogaster* PDFR-A C-terminal contains 28 Ser, Thr, or Tyr residues (these may be subject to post-translational phosphorylation and de-phosphorylation, and/ or other modifications). To survey putative functionality among these, we chose 14 residues that are distributed across the length of the C-terminal and which display high evolutionary conservation (**S1 Fig** and **S3 Table)**. For naming purposes, we grouped them into arbitrary clusters (CL) numbered #1 to #7, with positions shown in **Fig 1.** Following a common paradigm in study of GPCR physiology [e.g., 34–36], we performed an “Alanine Scan”: testing the consequences of their mutation to Alanine, which is a non-phosphorylatable analog [37]. Some clusters have only a single modified AA (e.g. CL4 and CL5), while in others we concurrently modified two AAs (e.g., CL1, CL6 and CL7), and in others, as many as six closely-positioned residues (e.g., CL2-3).

### Study overview

This series of variant receptors contains 10 different mutated versions of PDFR (not including the WT ‘parent’ PDFR-A) and is arbitrarily divided into two broad categories as indicated in **Fig 1**. The first group (termed “Simple Variants”) targeted one or two clusters of conserved Ser/Thr/Tyr AAs (i.e., CL 2-3A; 4A; 5A; 6A; 7A). The second category (termed “Multiple Variants”) targeted three or more of the AA clusters in various combinations (i.e., CL 1-4A; 1-5A; 1-6A; 1-7A and 5-7A). The CL1-7A variant is the most severe as it mutates all 14 of the targeted AA residues. We studied these ten PDFR variants, as well as the WT receptor, following expression in a *pdfr* mutant strain (*han*^*5537*^ [11]; we measured the ability of individual PDFR variants to rescue and to shape the phases and periodicity of rhythmic locomotor behavior. For the most part, we analyzed PDFR variants in the *pdfr* mutant background to permit evaluation of their properties, without competition from endogenous PDFR. Normally, PDFR-A is expressed throughout the ~150 cell pacemaker network, typically in subsets of each clustered group, as revealed by its expression from an epitope-marked BAC transgene [38]. However, no simple *Pdfr*-Gal4 driver element recapitulates a majority of normal expression sites [38]. Therefore to effect broad PDFR-A expression in the pacemaker system, we used a *timeless*-Gal4 driver element. Because PDF sensitivity varies with seasons [29], we tested this PDFR series in different photoperiodic conditions, as well as in constant darkness. *pdfr* loss-of-function mutants display advanced behavioral peaks under Light:Dark conditions, as well as weak and shortened free-running periods under constant darkness [11].

The behavioral actions of the Ala-mutated PDFRs either resembled those of WT receptor, or exhibited gain-of-function properties (behavioral actions opposite to those seen in loss-of-function *pdfr* mutant flies). For purpose of clarity, the Main Figures report a comparison of properties for the WT PDFR with those of the most extensively-mutated version of PDFR (called 1-7A), as indicated in **Fig 1**. Comparable data on the properties of the other variant receptors in the series is described in S1–S7 Figs. To better interpret the results produced by the different sequence variants, we also present experiments that test assumptions used in the experimental design. These tested the following hypotheses: i) that the behavioral effects of receptor variants are independent of activation by the endogenous ligand, neuropeptide PDF; and ii) that the bulky C-terminal fusion of GFP (present in all variants tested) strongly influenced the results. Both hypotheses were largely dis-proved.

### Effects of WT vs 1-7A PDFR on locomotor behavior in Short Day (winter-like) conditions (Fig 2)

**Fig 2:**
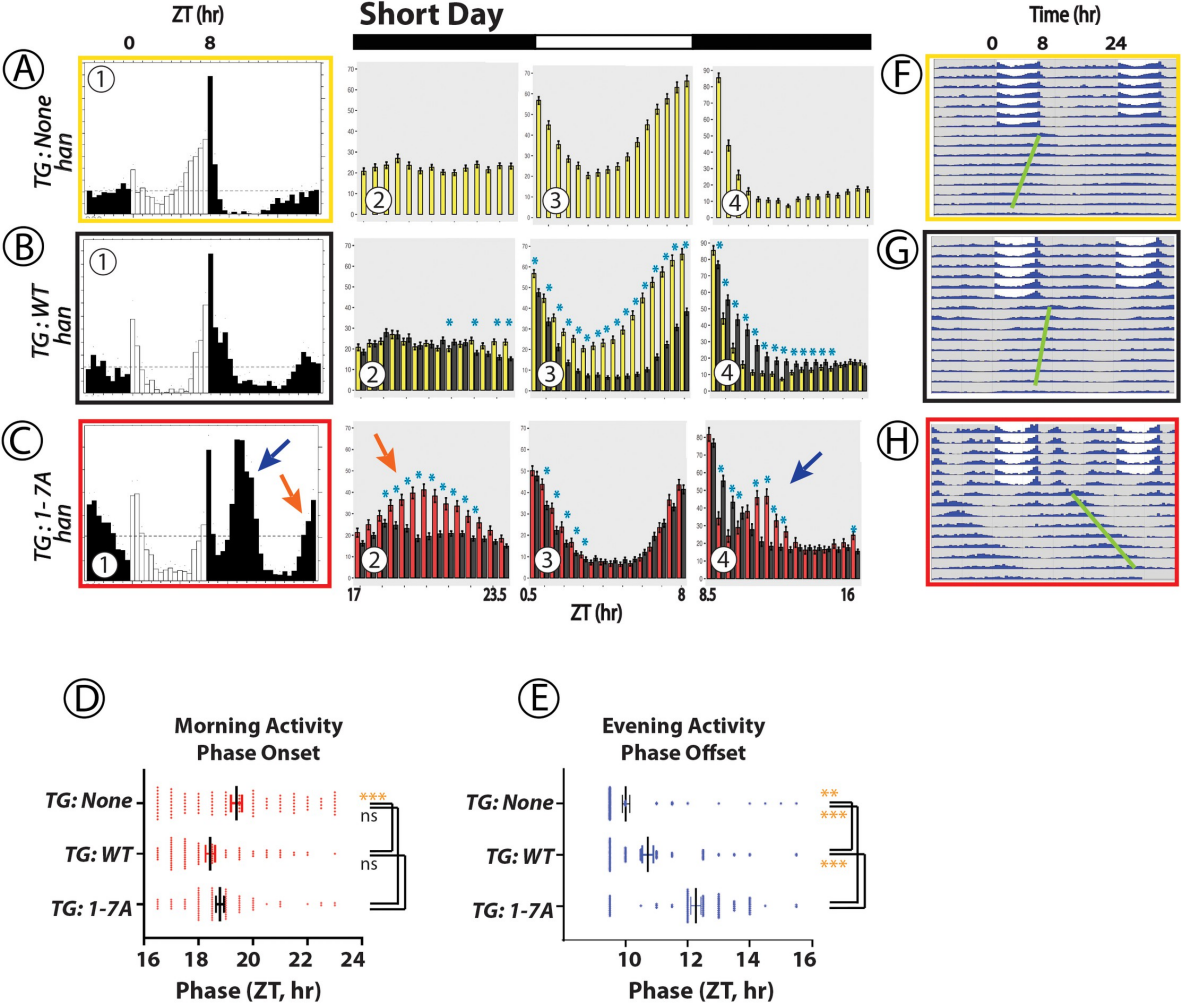
Locomotor Rhythms exhibited by *pdfr* mutant flies expressing the WT PDFR versus the 1-7A PDFR Variant under Winter-like (Short Day) conditions. All behavioral records were recorded from *han* (*pdfr* mutant) flies that expressed either no UAS transgene (A, yellow box), or a UAS-WT *pdfr* transgene (B, black box) or a UAS-1-7A *pdfr* (C, red box)). Panels (A)-(C) contains sub-panels 1–4. Sub-Panel (1) displays group eductions (6-day activity averages) with open bars indicating the 8-hr periods of Lights-on and filled bars the 16-hr periods of Lights-off. *TG*: transgene. The red and blue arrows indicate Morning and Evening activity periods respectively in the 1-7A records that have distinguished amplitude or phase. Amplitude measures are displayed in Panels 2–4: 30 min bins for each genotype, color-coded and directly-compared (i.e., Panel C-2 through C-4 compares the amplitudes of the WT PDFR activity (black) with that of the 1-7A PDFR activity (red). The bins representing the light-dark transitions were removed. Blue asterisks mark amplitudes that are significantly different between genotypes by ANOVA followed by a Students t-test (p < 0.05). Panel (D) displays the average Phase Onset timepoint for the Morning activity for each genotype over the last two days of entrainment (LD 5–6). Panel (E) displays the average Phase Offset timepoint for the Evening activity for each genotype over the last two days of entrainment (LD 5–6). Analyses in (D) and (E) represent ANOVA followed by Dunnett’s post hoc multiple comparisons of all compared to WT: ns = not significant; * = p<0.05; ** = p<0.01; *** = p<0.005; **** = p<0.001. Panels (F)–(H) present double plotted actograms of group activity for each genotype (color-coded) over the ~6 days of light entrainment, followed by ~9 days of constant darkness (DD). The green bars indicate the phases of the dominant activity periods in DD.

**Fig 3:**
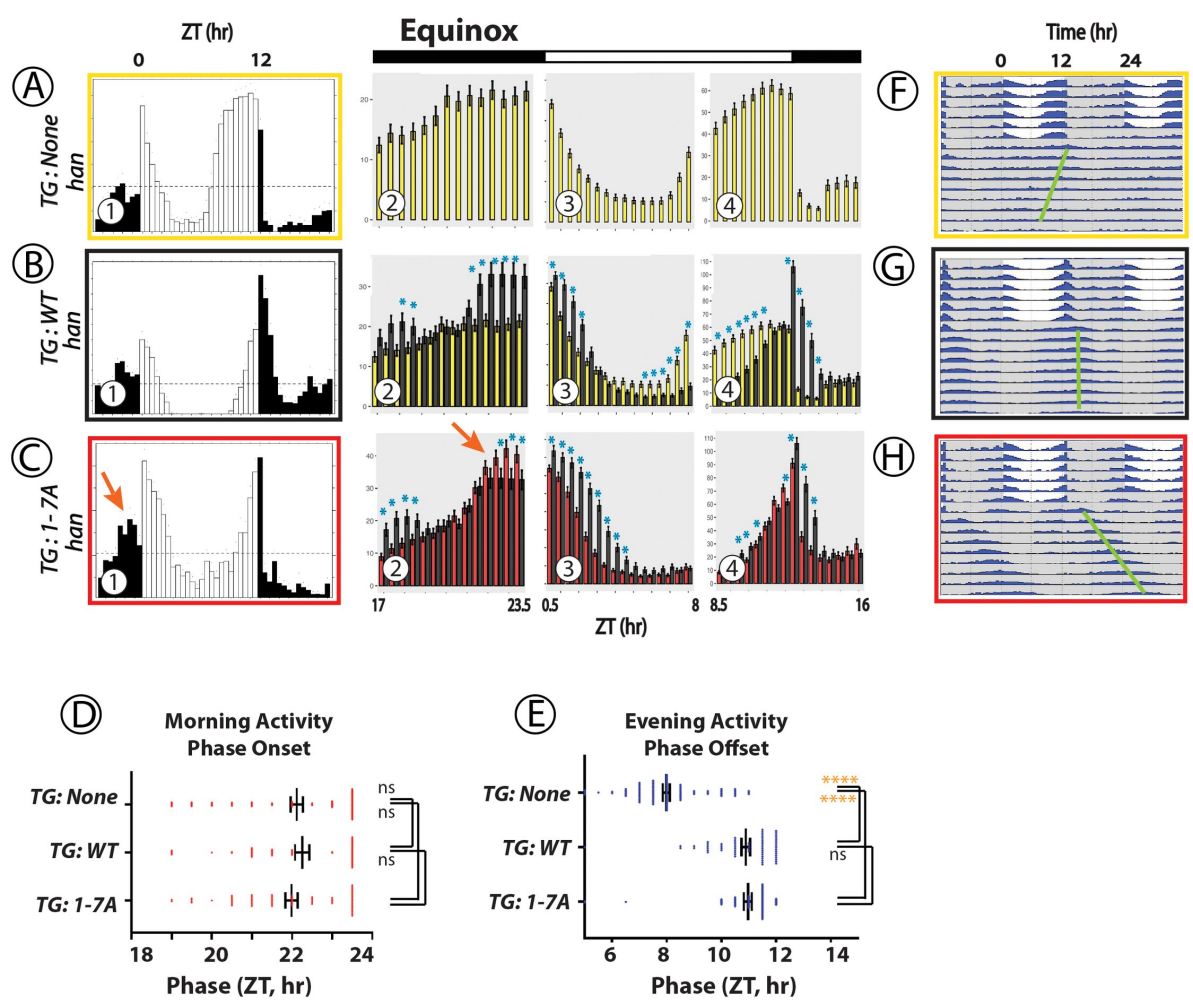
Locomotor Rhythms exhibited by *pdfr* mutant flies expressing the WT PDFR versus the 1-7A PDFR Variant under Equinox-like (12L:12D) conditions. All behavioral records were recorded from *han* (*pdfr* mutant) flies that expressed either no UAS transgene (A, yellow box), or a UAS-WT *pdfr* transgene (B, black box) or a UAS-1-7A *pdfr* (C, red box)). The Panels display behavioral activity with a format similar to the one shown in Fig 2. Panels (A-(C) display average group eductions (6-day activity averages, with open bars indicating the 12-hr periods of Lights-on and filled bars the 12-hr periods of Lights-off) and bin-by-bin analyses to compare activity amplitudes between genotypes. *TG*: transgene. The red arrow indicates Morning activity in flies expressing the 1-7A variant with distinguished amplitude. Blue asterisks mark amplitudes that are significantly different between genotypes by ANOVA followed by a Students t-test (p < 0.05). Panels (D) and (E) display the phases of morning Onsets and Evening Offsets respectively. Gold asterisks: ANOVA followed by Dunnett’s post hoc multiple comparisons of all compared to WT: ns = not significant; * = p<0.05; ** = p<0.01; *** = p<0.005; **** = p<0.001. Panels (F)–(H) present double plotted actograms of group activity for each genotype over the ~6 days of light entrainment, followed by ~9 days of constant darkness (DD). The green bars indicate the phase of the dominant activity period in DD. The green bars indicate the phases of the dominant activity periods in DD.

**Fig 4:**
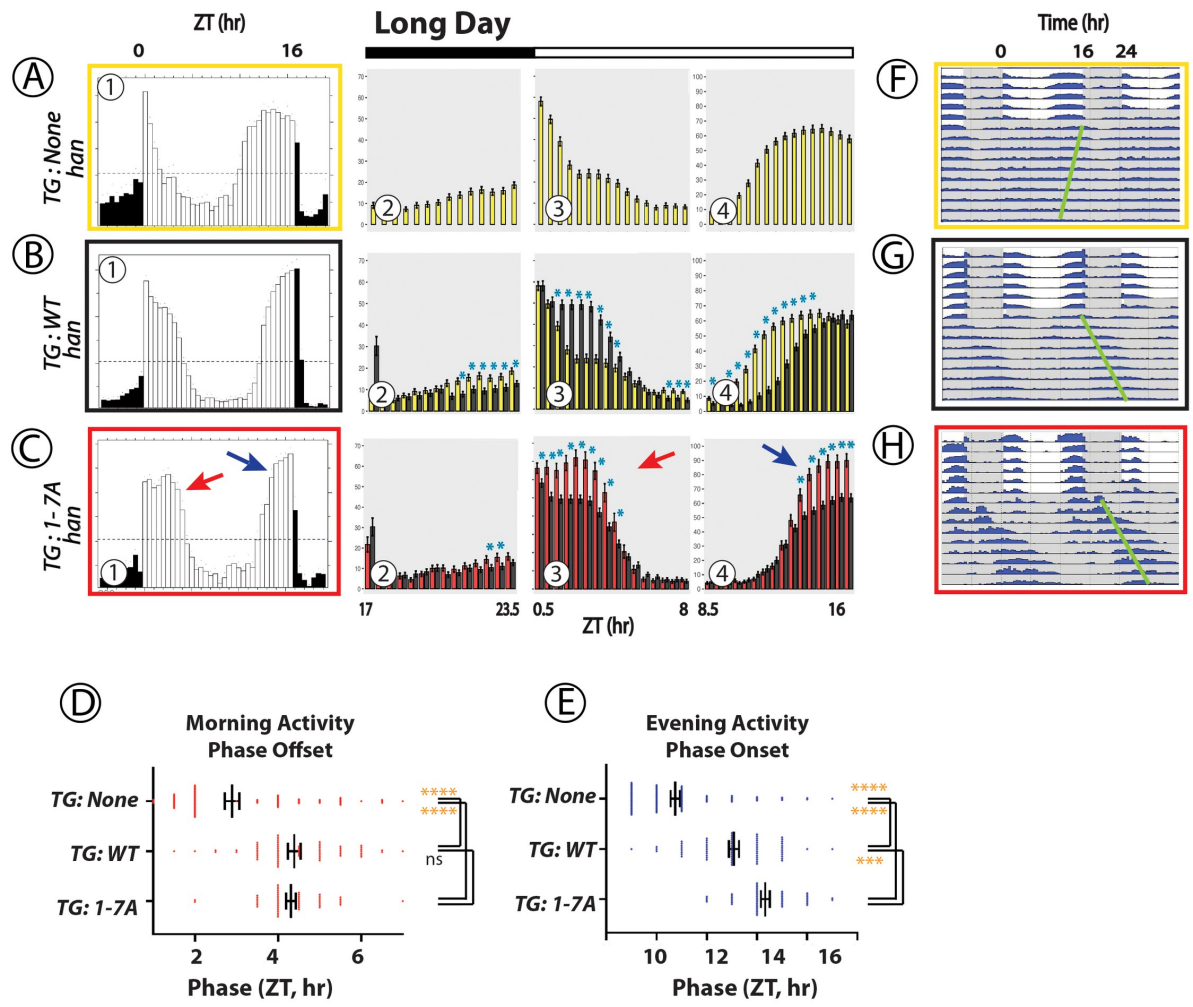
Locomotor Rhythms exhibited by *pdfr* mutant flies expressing the WT PDFR versus the 1-7A PDFR Variant under Long-day (summer-like) conditions. All behavioral records were recorded from *han* (*pdfr* mutant) flies that expressed either no UAS transgene (A, yellow box), or a UAS-WT *pdfr* transgene (B, black box) or a UAS-1-7A *pdfr* (C, red box)). The Panels present behavioral activity with a format similar to the one shown in Fig 2. Panels (A)-(C) display average group eductions (6-day average activity, with open bars indicating the 16-hr periods of Lights-on and filled bars the 8-hr periods of Lights-off) and bin-by-bin analyses to compare activity amplitudes between genotypes. *TG*: transgene. The red and blue arrows indicate Morning and Evening activity respectively, in flies expressing the 1-7A variant with distinguished amplitude and/or phase. Blue asterisks mark amplitudes that are significantly different between genotypes by ANOVA followed by a Students t-test (p < 0.05). Panels (D) and (E) display the phases of Morning Offsets and Evening Onsets respectively. Gold asterisks: ANOVA followed by Dunnett’s post hoc multiple comparisons of all compared to WT: ns = not significant; * = p<0.05; ** = p<0.01; *** = p<0.005; **** = p<0.001. Panels (F)–(H) display double plotted actograms of activity during the ~6 days of light:dark conditions, followed by activity during ~9 days of DD. The green bars indicate the phase of the dominant activity period in DD.

In 8L:16D, *pdfr*^*han*^ mutant flies lack a prominent morning peak and their evening peak of activity begins 1–2 hr earlier than controls; the example shown in **Fig 2A–1** (*tim*>*no transgene*, boxed in yellow) is heterozygous for the *tim*-Gal4 element. Rescue by a WT-*pdfr* cDNA (**Fig 2B–1** (*tim*>*pdfr*, boxed in black)) did not produce obvious effects on activity in the time domain preceding or just past Lights-On (Morning). However, it strongly affected the Evening peak: not its amplitude (**Figs 2B–3 and -4**) but significantly delaying its phase by about 1 h (**Fig 2E**) such that the Evening peak now extends past after Lights-off (**Fig 2B–4**). In contrast, expression of the 1-7A Multiple Variant driven by *tim*-Gal4 (*tim> pdfr1-7A*, boxed in red) elevated the amplitude of activity during the period of ZT 18–21 prior to lights-ON (**Fig 2C–1 and 2C-2, orange arrow**): we speculate this is promotion of a “Morning” peak of activity. It has the same phase as that produced by expression of the WT receptor (**Fig 2D**), but significantly larger amplitude (**Fig 2C2, orange arrow**). The 1-7A variant also delayed the Evening peak but to a much greater extent than did the WT PDFR (**Fig 2C–1**, **C-4**, blue arrow), producing a conspicuous, large amplitude peak that occurred on average as late as ~3 h after lights-off, at ZT12, significantly more delayed than the peak produced by WT PDFR (**Fig 2E**). Following short day light entrainment, flies were released into constant darkness for ~7–8 days (DD—constant conditions): resulting locomotor activity is displayed in the double-plotted group actograms (**Fig 2F—**yellow box**, G—**black box, and **H—**red box). The properties of the persistent circadian rhythmic behavior are provided in **Table 1**. The period (tau) of the 1-7A variant was considerably lengthened. The phase of the major DD rhythm matched that of the much-delayed evening peak in the 1-7A flies (**Fig 2H**, green line). In sum, under winter-like photoperiods, the PDFR 1-7A variant significantly increased the amplitude of the morning activity peak and significantly delayed the phase of the evening peak; the latter phase was also reflected in persistent rhythmic activity under subsequent DD.

### Behavioral effects of the other PDFR Variants under Short Day (winter-like) conditions)

We tested each of the nine other PDFR Ala-variants by comparing their effects on locomotor behavior as we had done for the PDFR 1-7A variant. **S2 Fig** presents these collected data with Group eductions, Actograms and Phase analysis; **S3 Fig** presents these same collected data with bin-by-bin comparisons to WT-PDFR to consider amplitude changes. The phase of the Morning peak was not obviously or systematically effected (**S2 Fig, panel L**), while that of the Evening peak was significantly delayed by several (**S2 Fig panel M**). We note two features–first the progressive addition of more Ala substitutions in the series 1-4A, 1-5A, 1-6A (**S2 Fig, panels I to K**) tended to produce large and delayed Evening peaks in the time period ZT11.5–12.5 (3–4 h after lights-off), with the variant 1-6A producing the most pronounced delay. The delayed Evening peak activity at ZT12 sometimes appeared at the expense of, the normal Evening peak activity that occurred prior to Lights-off (e.g., **S2 Fig, panels E to I and F to I**), although when averaged across all days in LD, that effect for specific variants was not significant (**S3 (E2 and F2) Fig**). Second, a delayed Evening phase was also produced by two of the Simple PDFR variants (**S2 Fig, panel M**), the 6A and 7A variants, each of which contain only a pair of Ser-to-Ala substitutions. The amplitude of the Morning peak (ZT18-20) was increased modestly by variants 2-3A, 7A and 1-5A (**S3 Fig, panels C-1, G-1 and J-1, red arrows**) or strongly by 6A (**S3 Fig, panel F-1, red arrow**). The amplitude of the Evening peak was also increased by others in the PDFR variant series (**S3 Fig, blue arrows**), of which the 6A, 7A, 1-5A and 1-6A variants had effect sizes most similar to that of 1-7A (**S3 Fig, panels F-3, G-3, J-3 and K-3).** We also note that combining the Single 5A, 6A and 7A variants into a Multiple Variant (5-7A) did not produce the anticipated additive effects on increasing Morning peak amplitude at ~ZT19 (**S3 Fig, panel H-1),** nor on increasing Evening peak amplitude **(S3 Fig, panel H-3).** Such anticipation was predicated on the effects seen individually with the 1-5A, 1-6A and 1-7A variants (versus the 1-4A); instead the 5-7A produced only a modest increase in the size of the delayed Evening peak **(S3 Fig, panel H-3).**

### Effects of WT versus 1-7A PDFR on locomotor behavior under equinox conditions (Fig 3)

Under 12:12 conditions, *han* mutant flies (lacking *pdfr* function) typically display elevated nocturnal activity, a lack of morning anticipation prior to Lights-on, and a pronounced advance in the peak of the Evening behavior [11]. Although we note that some reports have observed remnants or a full bout of Morning activity in *han* mutant flies [e.g., 37]. **Fig 3A–1** displays activity patterns of *han* mutants that are heterozygous for *tim*-Gal4; they generally matched prior descriptions of *pdfr* mutant behavior. Restoration of *pdfr* function by *han*; *tim*> WT-*pdfr*, restored a Morning peak (anticipatory activity prior to lights-on–**Fig 3B–1 and -2**) and delayed the evening peak by 1–2 hrs (**Fig 3B–1 and B-4**) [7,8,9]. Restoration of *pdfr* function by the 1-7A PDFR variant under these conditions (**Fig 3C–1**) increased the amplitude of the Morning activity peak but not its phase (**Fig 3C–2, red arrow**) but not its phase (**Fig 3D**). 1-7A expression in equinox conditions rescued the advanced phase of the Evening activity peak, but did not further delay it past Lights-OFF (**Fig 3C–4** and **3E**), unlike what we observed under short-day conditions. The amplitude of the Evening peak was only modestly elevated following expression of WT or 1-7A PDFRs. The period (tau) of the 1-7A variant was considerably lengthened, but see discussion below about the influence of the GFP fusion. The phase of the major DD rhythm was delayed relative to the evening peak under entraining conditions (**Fig 3H**, green line). In sum under equinox-like photoperiods, the PDFR 1-7A variant significantly increased the amplitude of the morning activity peak compared to WT PDFR, but did not differentially affect its phase, or the amplitude or phase of the Evening peak; under subsequent DD, the rhythmic activity displayed a delayed phase starting within the very first cycle.

### Behavioral effects of the other PDFR Variants under equinox conditions

**Fig 5:**
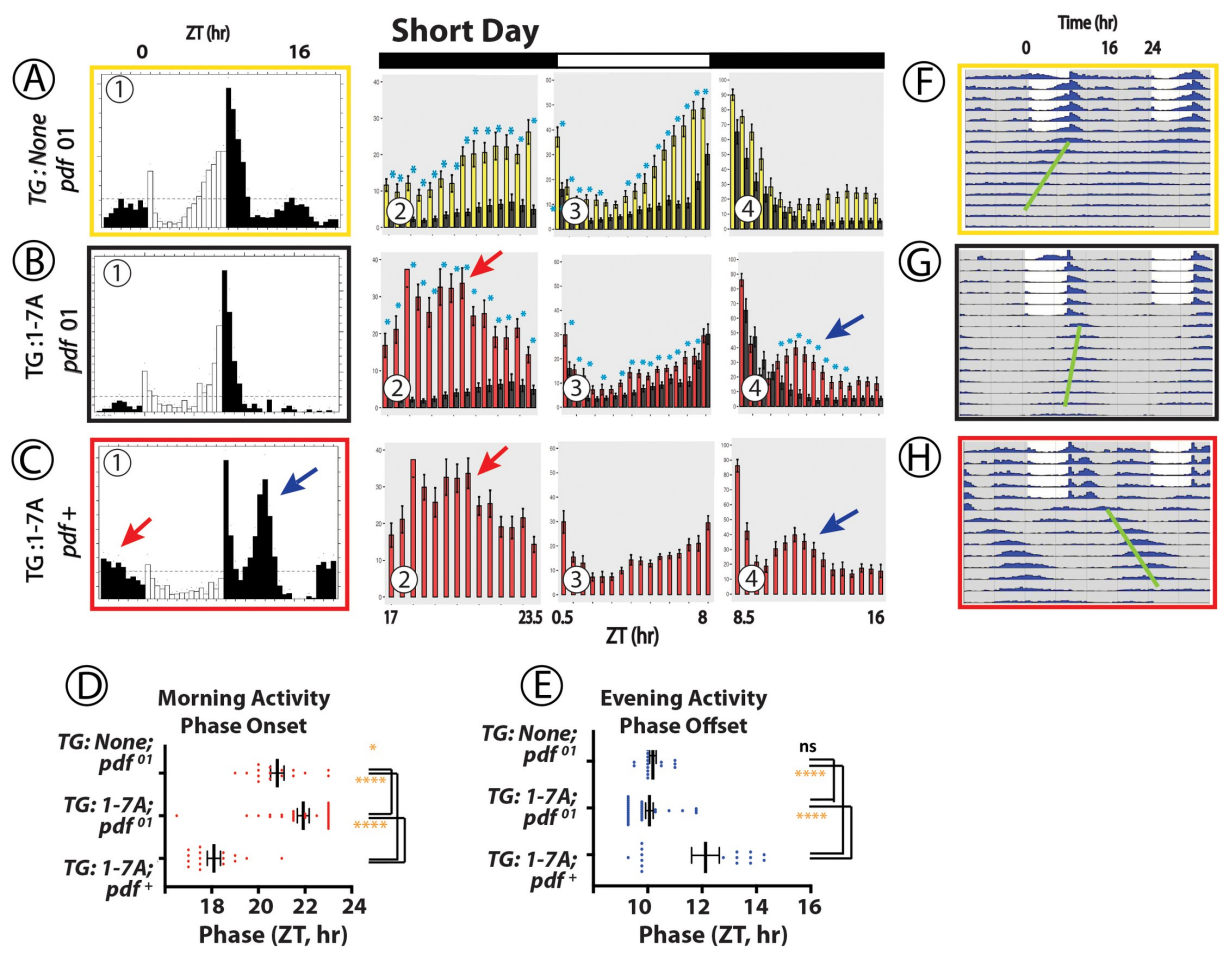
Locomotor Rhythms exhibited by WT versus *pdf* mutant flies expressing the 1-7A PDFR Variant under Short-day (winter-like) conditions. The Panels present behavioral activity with a format similar to that described in Figure Legend 2. Behavioral records were recorded from *pdf*^*01*^ (null) flies that expressed either no UAS transgene (A, yellow box), or a UAS-1-7A *pdfr* transgene (B, black box), or from control flies (*pdf*^*+*^*)* that expressed the UAS-1-7A *pdfr* (C, red box)). *TG*: transgene. Panels (A)-(C) display average group eductions (6-day average activity, with open bars indicating the 8-hr periods of Lights-on and filled bars the 16-hr periods of Lights-off) and bin-by-bin analyses to compare activity amplitudes between genotypes. The red and blue arrows indicate Morning and Evening activity of control (red) flies with distinguished amplitude and/or phase, respectively. Blue asterisks mark amplitudes that are significantly different between genotypes by ANOVA followed by a Students t-test (p < 0.05). Panels (D) and (E) display the phases of Morning activity Onsets and Evening activity Offsets respectively. Gold asterisks: ANOVA followed by Dunnett’s post hoc multiple comparisons of all compared to WT: ns = not significant; * = p<0.05; ** = p<0.01; *** = p<0.005; **** = p<0.001. Panels (F)–(H) display double plotted actograms of activity during the ~6 days of light:dark conditions, followed by activity during ~9 days of DD. The green bars indicate the phase of the dominant activity period in DD.

We tested each of the nine other PDFR Ala-variants by comparing their effects on locomotor behavior as we had done for the PDFR 1-7A variant. The phases of the Morning and Evening peaks were not obviously or systematically effected (**S4 Fig, panels L** and **M**). Relative to WT PDFR expression, the Morning Activity peak amplitude was increased by expression of the 6A, 7A and 1-5A variants (**S5 Fig, panels F-1, G-1**, and **J-1**, red arrows). Relative to WT PDFR expression, the Evening Activity peak amplitude was increased by expression of the 4A, 6A, 7A, 5-7A and 1-5A variants (**S5, Fig 5, panels D-4, F-4, G-4**, **H-4** and **J-4**, blue arrows). We also note that combining the Single 5A, 6A and 7A variants into a Multiple Variant (5-7A) did not produce the anticipated additive effects on increasing Morning peak amplitude at ~ZT21 (**S5 Fig, panels H-1, versus 5F-1 and G-1).**

### Behavior under long day (summer) conditions (Fig 4)

Flies lacking *pdfr* function in 16L:8D conditions display locomotor patterns similar to those in 12L:12D –lack of a clear Morning peak and display of a broad Evening activity period that peaks ~2-3hr before Lights-off: the example in **Fig 4A–1 (yellow box)** displays behavior by *han* mutants that are also heterozygous for the *tim*-Gal4 element. Rescue (*han*; *tim*> WT-*pdfr*, **Fig 4B–1, black box**) typically delayed Morning activity offset (**Fig 4B–3 and 4D)** and delayed the Evening peak onset each by 1.5 h (**Fig 4B–4 and 4E).** Relative to that of WT PDFR, expression of the 1-7A variant (**Fig 4C–1**) increased the amplitude of both the morning activity peak (**Fig 4C–3**) and the evening activity peak (**Fig 4C–4**). It did not significantly alter the Morning phase relative to effects of WT PDFR (**Fig 4D),** although there was a pronounced tendency to broaden the duration of the Morning activity peak. 1-7A expression did significantly extend the delay the Evening phase of activity relative the effect of Wt PDFR **(Fig 4E**). In DD, the 1-7A PDFR variant significantly lengthened circadian period and the dominant rhythmic activity displayed a phase several hours delayed from the evening activity peak phase under light entraining conditions within the first cycle (**Fig 4H**–green bar). In summary the 1-7A PDFR variant significantly increased the amplitudes of the Morning and Evening activity peaks under long days, and also slightly delayed the Evening activity phase.

### Behavioral effects of the other PDFR Variants under long day (summer-like) conditions

We tested each of the nine other PDFR Ala-variants by comparing their effects on locomotor behavior as we had done for the PDFR 1-7A variant. There was no systematic effect of the variants on the phase of the Morning peak (**S6 Fig, panel L**), but several did delay the Evening activity phase (**S6 Fig, panel M**), especially the 1-4A, 1-5A and 1-6A variants. A few variants affected the Morning peak amplitude– 5A, 6A and 1-6A (**S7 Fig, panels E-2, F-2 and K-2**). Several increased the amplitude of the Evening peak under these conditions, namely 2-3A, 4A, 5A, 6A, 7A, 1-5A and 1-6A (**S7 Fig, panels C-4, D-4, E-4, F-4, G-4, J-4** and **K-4**). As observed in the other photoperiodic conditions, when we combining the 5A, 6A and 7A variants into a single 5-7A variant, it did not produce the expected additive effects on behavior in the morning (**S7 Fig, panel 7H-2**) or in the evening (**S7 Fig, panel H-3**): instead these activities appeared similar to effects displayed by the 1-4A variant.

### Effects of PDFR variants on period and phase of rhythmic activity in DD

In DD, following Short Day conditions, the PDFR variants that produced 3–4 h delays in the evening peak often generated periods ~ 1–2 hr longer than the controls and also lowered % arrhythmicity (**Table 1**). This was especially true for the Multiple Variant series (e.g., PDFR 1-5A, 1-6A and 1-7A (**Table 1**). However the correlation between delayed evening peaks and a longer tau in DD was not absolute. For example, the 6A, 7A and 5-7A variants all had strong and significant delaying effects on the Evening phase of activity under Short Day conditions (compared to the effect of the WT PDFR), but they did not lengthen tau values in subsequent DD conditions (**Table 1**). These observations suggest that delays of activity phases produced by PDFR modulation in LD may not reflect a direct consequence of PDFR effects on the PER and TIM—dependent clock [2,3,17]. In addition, the dominant activity periods in DD typically reflected the delayed (~ZT12) Evening peak (**S2 Fig. panels F-2, 2G-2** and **2H-2**). Notably, the early Morning peak (~ZT18-19) promoted by PDFR 6A and 7A variants clearly persisted in DD (**S2 Fig, panels F-2, G-2**). In DD following Equinox or Long Day entrainment (with photophases > 8 hr in duration), we noted that several variants produced dominant activity phases which were phase-delayed relative to the phase of the Evening peak displayed in LD (e.g., 6A and 7A –**S4 Fig, panels F-2 and G-2**;. **S6 Fig, panels F-2 and G-2).** Again, several of the variants significantly increased Tau in DD (**Table 1**), yet had no delaying effects on phase in LD: examples included 2-3A, 1-4A, and 1-5A, under equinox conditions (**S4 Fig, panels C-2, I-2, J-2**) and 4A and 1-5A in long day conditions (**S6 Fig, panels. D-2, 6J-2).**

**Table 1:** Locomotor activity measures of various genotypes during constant dark conditions.

						Average tau different from:		
Short Day Entrainment	N	n	%AR	tau	SEM	w[1118]	pdfrEgfp	pdf^01^
*han[5304]; tim > w[1118]*	5	72	22%	23.9	0.09	-	ns	nd
*han[5304]; tim > pdfr*	1	16	6%	23.7	0.13	ns	ns	nd
*han[5304]; tim > pdfr1-7A No GFP*	3	30	8%	23.6	0.07	ns	ns	nd
*han[5304]; tim > pdfrEgfp*	3	46	35%	24.0	0.09	ns	-	nd
*han[5304]; tim > pdfrEgfp23A*	2	31	13%	24.8	0.12	ns	ns	nd
*han[5304]; tim > pdfrEgfp4A*	2	31	26%	24.7	0.13	ns	ns	nd
*han[5304]; tim > pdfrEgfp5A*	2	43	26%	24.6	0.11	ns	ns	nd
*han[5304]; tim > pdfrEgfp6A*	3	50	38%	24.4	0.08	ns	ns	nd
*han[5304]; tim > pdfrEgfp7A*	3	45	27%	24.8	0.11	ns	ns	nd
*han[5304]; tim > pdfrEgfp1-4A*	3	40	12%	24.6	0.12	ns	ns	nd
*han[5304]; tim > pdfrEgfp1-5A*	3	31	16%	25.0	0.23	***	*	nd
*han[5304]; tim > pdfrEgfp1-6A*	3	42	14%	25.1	0.13	****	**	nd
*han[5304]; tim > pdfrEgfp1-7A*	4	59	16%	24.9	0.11	****	*	nd
*han[5304]; tim > pdfrEgfp567A*	2	37	16%	23.9	0.09	ns	ns	nd
*tim > pdfrEgfp1-7A*	1	15	0%	25.8	0.25	nd	nd	****
*pdf^01^*	1	12	67%	22.5	0.12	nd	nd	-
*tim > pdfrEgfp1-7A; pdf^01^/pdf^01^*	1	25	20%	23.6	0.12	nd	nd	ns
**Equinox Entrainment**	**N**	**n**	**%AR**	**tau**	**SEM**			
*han[5304]; tim > w[1118]*	6	68	34%	23.7	0.09	-	ns	
*han[5304]; tim > pdfr*	3	16	25%	23.5	0.16	ns	ns	
*han[5304];; tim > pdfr1-7A No GFP*	3	35	14%	23.7	0.08	ns	ns	
*han[5304]; tim > pdfrEgfp*	4	48	13%	24.1	0.06	ns	-	
*han[5304]; tim > pdfrEgfp23A*	2	54	48%	25.6	0.12	****	****	
*han[5304]; tim > pdfrEgfp4A*	2	44	32%	24.1	0.07	ns	ns	
*han[5304]; tim > pdfrEgfp5A*	2	47	34%	24.4	0.06	ns	ns	
*han[5304]; tim > pdfrEgfp6A*	3	35	23%	24.3	0.13	ns	ns	
*han[5304]; tim > pdfrEgfp7A*	3	47	21%	24.1	0.09	ns	ns	
*han[5304]; tim > pdfrEgfp1-4A*	3	45	18%	24.5	0.08	**	ns	
*han[5304]; tim > pdfrEgfp1-5A*	2	32	6%	25.0	0.13	****	***	
*han[5304]; tim > pdfrEgfp1-6A*	2	32	19%	25.3	0.14	****	****	
*han[5304]; tim > pdfrEgfp1-7A*	6	88	6%	25.1	0.17	****	****	
*han[5304]; tim > pdfrEgfp567A*	2	45	24%	24.1	0.08	ns	ns	
**Long Day Entrainment**	**N**	**n**	**%AR**	**tau**	**SEM**			
*han[5304]; tim > w[1118]*	7	###	47%	23.8	0.06	-	ns	nd
*han[5304]; tim > pdfr*	3	47	17%	24.9	0.15	ns	ns	nd
*han[5304]; tim > pdfr1-7A No GFP*	3	40	48%	24.6	0.09	ns	ns	nd
*han[5304]; tim > pdfrEgfp*	4	82	17%	24.9	0.08	ns	-	nd
*han[5304]; tim > pdfrEgfp23A*	2	48	23%	25.4	0.12	****	****	nd
*han[5304]; tim > pdfrEgfp4A*	1	27	22%	25.5	0.16	****	****	nd
*han[5304]; tim > pdfrEgfp5A*	1	36	11%	24.7	0.16	ns	ns	nd
*han[5304]; tim > pdfrEgfp6A*	2	51	22%	25.0	0.09	ns	ns	nd
*han[5304]; tim > pdfrEgfp7A*	2	54	13%	25.5	0.09	****	****	nd
*han[5304]; tim > pdfrEgfp1-4A*	3	40	8%	25.2	0.13	****	ns	nd
*han[5304]; tim > pdfrEgfp1-5A*	3	33	6%	25.2	0.14	****	*	nd
*han[5304]; tim > pdfrEgfp1-6A*	3	48	2%	25.6	0.09	****	****	nd
*han[5304]; tim > pdfrEgfp1-7A*	4	63	14%	25.3	0.08	****	***	nd
*han[5304]; tim > pdfrEgfp567A*	1	38	18%	24.3	0.09	ns	ns	nd
*tim > pdfrEgfp1-7A*	1	14	14%	25.5	0.13	nd	nd	****
*pdf^01^*	1	32	88%	22.7	0.05	nd	nd	-
*tim > pdfrEgfp1-7A; pdf^01^/pdf^01^*	1	29	21%	23.3	0.09	nd	nd	ns

Behavioral activity records from days 3–9 in constant conditions following entrainment in the three indicated photoperiods (Short Day, Equinox and Long Day). N = number of independent experiments performed for each genotype::photoperiod combination. n = total number of flies tested for each genotype::photoperiod combination. % AR: the percentage of flies judged arrhythmic by criteria; tau = average circadian periods calculated according to χ2-periodogram analysis. Statistical analysis compared tau’s using Tukey’s multiple comparisons post hoc test following a one-way ANOVA: ns: not significant;

* p < 0.05

** p < 0.01

*** p < 0.001

**** p < 0.0001.; nd: not determined.

### Control experiments

**Fig 6:**
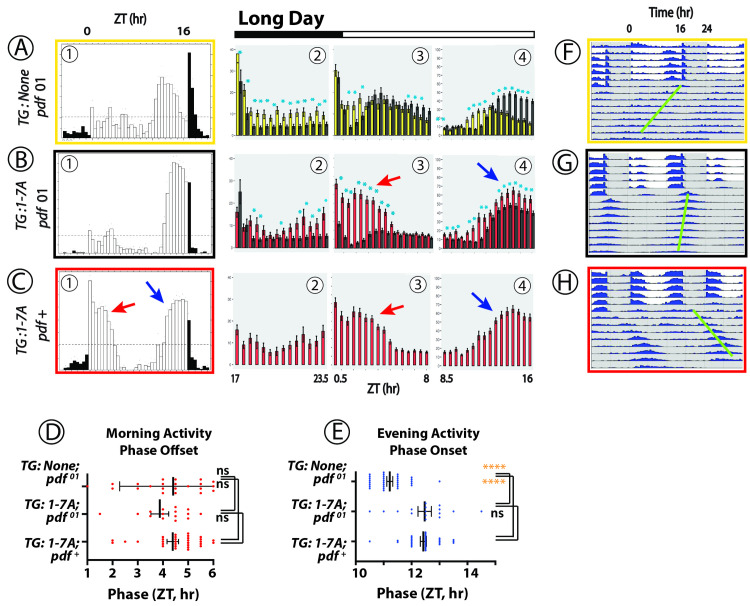
Locomotor Rhythms exhibited by WT versus *pdf* mutant flies expressing the 1-7A PDFR Variant under Long-day (summer-like) conditions. The Panels present behavioral activity with a format similar to that described in Figure Legend 5. Behavioral records were recorded from *pdf*^*01*^ (null) flies that expressed either no UAS transgene (A, yellow box), or a UAS-1-7A *pdfr* transgene (B, black box), or from control flies (*pdf*^*+*^*)* that expressed the UAS-1-7A *pdfr* (C, red box)). *TG*: transgene. Panels (A)-(C) display average group eductions (6-day average activity, with open bars indicating the 16-hr periods of Lights-on and filled bars the 8-hr periods of Lights-off) and bin-by-bin analyses to compare activity amplitudes between genotypes. The red and blue arrows indicate Morning and Evening activity of control (red) flies with distinguished amplitude and/or phase, respectively. Blue asterisks mark amplitudes that are significantly different between genotypes by ANOVA followed by a Students t-test (p < 0.05). Panels (D) and (E) display the phases of Morning activity Onsets and Evening activity Offsets respectively. Gold asterisks: ANOVA followed by Dunnett’s post hoc multiple comparisons of all compared to WT: ns = not significant; * = p<0.05; ** = p<0.01; *** = p<0.005; **** = p<0.001. Panels (F)–(H) display double plotted actograms of activity during the ~6 days of light:dark conditions, followed by activity during ~9 days of DD. The green bars indicate the phase of the dominant activity period in DD.

The design of the *PDF*R variants contains assumptions and genotypic constraints, distinct from simply substituting Alanine at potential sites of phosphorylation in the receptor C terminal tail. To assess the potential of some assumptions to affect the results we report, we performed the following two sets of experiments as controls. ***Control experiments (i)—dependence of GPCR PDFR variant effects on the presence of PDF ligand***. We assumed that the altered behavioral phenotypes produced by certain PDFR variants depended on activation by their endogenous cognate ligand, the neuropeptide PDF. However, we could not *a priori* exclude the possibility that PDFR variants may in fact produce novel constitutive activity (neomorphic properties). We therefore placed the 1-7A PDFR variant in the *pdf*^*01*^ (null) background and re-tested its activity when driven by *tim*(UAS)-Gal4 under both short day and long day conditions, and then in constant darkness. In all conditions, the behavioral phenotypes largely resembled those of the *pdf*^*01*^ background (lack of a morning activity peak, advanced evening peak, and shorter tau/s in DD). The results are shown in **Fig 5** (under short days) and **Fig 6** (under long days) and tabulated in **Table 1**. The ability of the 1-7A PDFR variant to increase the amplitude of the Morning peak was completely dependent on WT *pdf* function (**Fig 5B–2**). Likewise its ability to significantly delay the Evening phase into the dark period was completely dependent on WT *pdf* function (**Fig 5B–4 and 5E**). Under Long days, the ability of the 1-7A variant to increase the amplitudes of the Morning and Evening activity peaks were strongly diminished by lack of WT pdf function (**Fig 6B–3** and **B-4**). The 1-7A variant did display some activity in the *pdf* mutant background: for example, it produced a significant delay of the Evening peak phase (akin to the action of the WT receptor, **Fig 5A–3** and **Fig 6 A-4**) and a reduction in the % arrhythmicity to nearly the same value as found in a WT *pdf* background. These results suggest some degree of constitutive (ligand-independent) activity. Apart from these, we conclude that constitutive activity explains at best a small proportion of the behavioral effects that distinguish the 1-7A PDFR variant from the WT receptor.

(i)—***The influence of epitope fusions*.** The second control experiment asked whether the 1-7A epitope fusions (4xFLAG and eGFP), might confer some of the differences in behavioral effects that we observed. In part, the concern regarding fused sequences is mitigated by the fact that all UAS-*PDFR* isoforms in the experimental series contained both epitopes (in addition–all were genetically transduced to the same integration site). Hence comparisons across different variants should largely normalize for the effects of the non-receptor sequences, and instead highlight variant-specific properties. However, we wished to test this assumption explicitly to define the extent to which a bulky GFP fused to the C Terminal tail might confer some of the behavioral properties we might otherwise ascribe to PDFR C terminal sequences. We therefore compared a 1-7A version of PDFR to a wild type PDFR, both of which lacked GFP fusions. We found that the additional Evening phase delays in short day and long day produced by 1-7A expression were present in the absence of the GFP fusion (**S8 Fig, panels B-4, C-4, H** and **J-4 and P**); in addition, the increase of the Morning amplitude under Long days was present in the absence of the GFP fusion (**S8 Fig, panel J-3**). Finally, the phase of the main DD rhythmic activity, following expression of 1-7A lacking a GFP fusion, reflected the delayed evening phase in short day (**S8 Fig, panel F**, green bar) and was delayed relative to that of the LD Evening activity peak in long days (**S8 Fig, panel N**, green bar). We note however that the longer period produced by the 1-7A variant was not exhibited by the 1-7A lacking GFP, suggesting that a lengthened period largely depends on the GFP fusion. In sum, these results support the hypothesis that most of the key alterations in daily locomotor rhythmicity derived from sequence variation of the PDFR, and not from the properties of the fused GFP.

### PDFR expression and signaling *in vitro*

The PDFR is a G_s_-coupled receptor and several reports have documented the importance of the downstream cAMP pathway to mediate PDF behavioral regulation [15, 17,39]. In that context, we asked whether the *in vivo* properties of those PDFR variants that affected behavioral phase and amplitude could be correlated with *in vitro* properties when expressed in *hEK-293T* calls. We found no strong correlation. Significant differences in basal signaling levels, in EC50 or in maximum values of signal transduction were rare (**S9 Fig, S10 Fig and S11 Fig**). When noted, they were poorly correlated with behavioral effects (**S5 Table**). We also measured surface expression of the PFDR variants in *hEK* cells using β-lactamase N terminal fusions [40] to determine if PDFR variants tended to display longer surface lifetimes. The 1–4 and 1–7 variants displayed higher basal levels, but no others were different from the WT levels (**S12 Fig**). Following 20 min exposure to PDF, neither the WT not any of the variants displayed a change in surface expression levels (**S13 Fig**).

### Measuring the phosphorylation state of over-expressed PDFR *in vivo*

To obtain direct evidence that PDFR sequences are phosphorylated *in vivo*, we first over-expressed an epitope-tagged- PDFR WT construct using *tim*-Gal4, which directs expression broadly in cells that feature the PER-dependent molecular oscillator. We immunoprecipitated the receptor from head extracts, then employed tandem mass spectroscopy to determine which if any specific PDFR peptide fragments are phosphorylated. We performed eight biological replicates, with two collections in the morning (ZT2-3) and six in the evening hours (ZT11). The **S4 Table** reports the phosphorylated peptides detected from the PDFR-GFP fusion protein. Among the 28 conserved Ser/Thr and Tyr residues in the PDFR C terminal tail, five were phosphorylated in one or more of these samples. In the two Mornings samples S563 was phosphorylated in one sample; in the Evening samples, S531 (in CL2) was phosphorylated in four of samples, S534 (in CL2) in one, T543 (in CL3) in one, and S560 in two of six samples. The spectra documenting detection of these phosphopeptides are presented in **S14 Fig**.

### Testing GRKs and β-arrestin2 contributions to locomotor rhythmicity

Regarding which potential PDFR phosphorylation sites might be β-arrestin binding sites, we considered a recent report by Zhou *et al*. [41], who proposed a conserved GPCR sequence motif that promotes high affinity interactions with β -arrestin. The motif includes three phosphorylation sites that align with three conserved, positively-charged pockets in the arrestin N-domain. In *D*. *melanogaster* PDFR, the CL1 region contains a match with the proposed motif beginning with S512 (SLATQLS) and shows moderate sequence conservation: of the 17 species we considered, 9 others retained this precise motif (**S1 Fig**). In addition, the *D*. *melanogaster* PDFR sequence beginning with S629 (SRTRGS) also displays a match for the motif, but is retained in only 6 of the other 16 species. The evidence that these two sites represent high affinity β-arrestin2 binding domains is therefore equivocal. We previously reported that β-arrestin2-GFP is not efficiently recruited to activated PDFR when functionally-expressed in *hEK-293T* cells [29]. In contrast, each of 13 other *Drosophila* neuropeptide GPCRs do efficiently recruit β-arrestin2-GFP when expressed and activated in that cellular environment [30–32].

We tested the potential to detect *in vivo* involvement by *Drosophila* orthologues of the canonical desensitization effectors—mammalian G-protein coupled receptor kinases (GRKs–GPRK1 (CG40129) and GPRK2 (CG17998)) and of mammalian β-arrestin2 (βarr2 –*kurtz* (CG1487))—in the control of locomotor rhythms We drove specific over-expression of WT cDNAs and RNAi constructs using *tim*-Gal4, to broadly affect signaling in the circadian pacemaker system. The PDFR-PA protein is highly restricted in its expression to subsets of the pacemaker neural network [9]. We predicted that elimination of a desensitizing component for PDFR signaling should produce behavioral phenotypes opposite to that of *pdfr* loss of function phenotypes: these would potentially include (for example) a delayed evening activity phase in LD conditions and a longer tau under DD. GPRK1- and *krz*-specific RNAi’s produced normal average locomotor profiles under 12:12, while one of two GPRK2-specific RNAi constructs tested slightly advanced the evening peak (**S15 Fig**). Over-expressing GPRK1 cDNAs did not affect morning or evening phase; over-expressing GPRK2 broadened the evening peak. Under DD, *krz* RNAi flies were uniformly arrhythmic, while GPRK RNAi’s were normal or slightly lengthened the circadian period (**S5 Table**). These results do not support the hypothesis, and suggest that the kinetics by which PDFR signaling terminates do not depend exclusively on the activities of either dGPRK-1 or -2, or on that of the *Drosophila* β-arrestin2 ortholog, *krz*.

## Discussion

We employed a gain-of-function approach to measuring neuropeptide GPCR function *in vivo*. In doing so, we sought to learn about the maximal consequences of PDFR GPCR signaling and also learn something about the phosphorylation events that normally control the duration of PDFR’s signaling lifetime. We found that expression of a PDF receptor variant, containing numerous Ala-substitutions of conserved phosphorylatable residues in the C terminal domain (1-7A), fundamentally altered rhythmic locomotor behavior in *Drosophila*. The changes included increases in the amplitudes of the Morning and/or Evening activity peaks, and delays in the Phase of the Evening peak. Such results are generally consistent with a prediction whereby non-phosphorylatable PDFR variants–those with a potential to increase the duration of PDFR signaling—would produce behavioral actions opposite to those seen in loss-of-function *pdf* [1], or *pdfr* [11] mutant stocks. Accordingly, we speculate that phosphorylation of the PDFR at these conserved residues normally defines the duration of its active signaling state each day. The longer duration that follows the substitutions of Ala may allow receptor signal strength to also increase by accrual: such an effect could explain the increased amplitude of the Morning and Evening peaks, as seen with expression of some of the variants. In this context, we acknowledge that modification of GPCRs by phosphorylation does not exclusively lead to signal termination. GRK2-dependent phosphorylation of the smoothened (smo) GPCR follows reception of the Hh signal and helps mediate its signal: phosphorylation leads to smo activation in both *Drosophila* and mammalian systems [42]. Thus modification of putative phosphorylation sites on GPCRs (to preclude phosphorylation) will not exclusively promote extended signaling. Therefore our interpretations must correspondingly consider outcomes without *a priori* assumption of mechanisms.

### GPCR signal termination

PDFR belongs to the Secretin Receptor Family (Family B) of neuropeptide receptors [11–13]: there is no clear consensus regarding mechanisms of desensitization and internalization for this receptor family. For VPAC2 receptors, phosphorylation and internalization is mediated exclusively by GRK2 [43]. Likewise, *Drosophila* orthologues of Cortocotrophin Releasing Factor receptors (CG8422, DH44-R1) and Calcitonin receptors (CG17415, DH31-R1) are internalized in *hEK* cells following recruitment of β-arrestin2 [31–32]. In contrast, PDFR, which is also related to the mammalian Calcitonin receptor, is not internalized following exposure to PDF [29]. VPAC2 receptors are also regulated by heterologous receptor signaling: M3 cholinergic receptors via PKC signaling can block VPAC2 phosphorylation, desensitization and internalization [44]. Furthermore, secretin receptors and VPAC1 receptors undergo phosphorylation by GRKs and β-arrestin2-dependent desensitization, but these are not sufficient to facilitate or mediate internalization [45–47]. Finally, GLP2-Receptor associates with β-arrestin2 via its distal C terminal sequences, but that receptor domain is required neither for GLP2-R desensitization nor its internalization [48]. Thus kinases other than GRKs and effectors other than β-arrestin2 may regulate internalization of diverse Family B receptors.

Following activation of rhodopsin in the mammalian retina, visual arrestin is recruited with a time constant of < 80 ms [49]; for many *Drosophila* neuropeptide receptors, β-arrestin2 is recruited within a minute of exposure to ligand [30]. The PDFR GPCR signals over a time base of many hours [19]: could phosphorylation/de-phosphorylation also regulate its activity? The mammalian blue-light sensitive GPCR melanopsin (OPN4) mediates intrinsic light sensitivity in certain classes of retinal ganglion cells (RGCs): melanopsin signaling is distinguished by long latencies, graded responses and sustained RGC depolarization that can outlast the duration of the light stimulus [50]. Melanopsin-expressing RGCs produce exceptionally long time-course integration because that GPCR behaves differently from other opsins in two fundamental ways. Firstly, it undergoes photoequilibration between signaling and silent states: a property that maintains the availability of pigment molecules for activation, termed ‘tristability’ by Emmanual and Do [51]. Secondly, phosphorylation and β-arrestin recruitment do contribute to the kinetics of melanopsin signaling, but over a long time-course [34,52]. Mure *et al*. [34] proposed that the distal portion of the melanopsin C terminal tail creates a steric blockade covering a cluster of specific serine residues situated in more proximal regions. The time required for relief of that blockade dictates the time course of GPCR phosphorylation and therefore delays subsequent desensitization.

### Specific versus non-specific phosphorylation regulating GPCR activity

**Fig 7:**
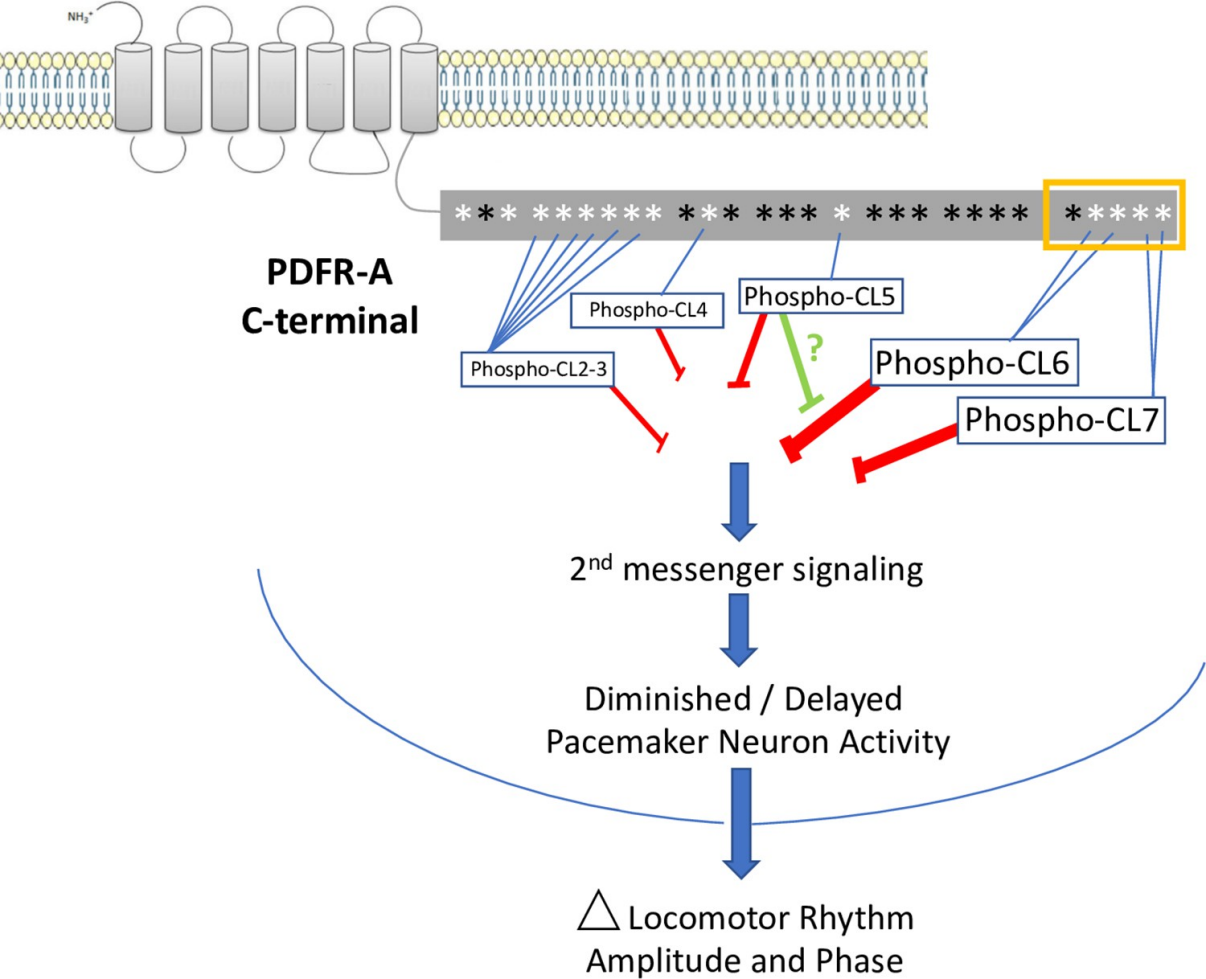
A model predicting the effects of phosphorylating different sites on the PDFR CT on downstream signaling. The CT is diagrammed as a grey box running horizontally. Asterisks indicate the positions Ser/ Thr/ and Tyr residues as described in **Fig 1** and **S1 Fig**: white asterisks are the subset of Ser/Thr/ and Tyr residues that were targets of mutational analysis in this study. Mutation of CL6 and CL7 consistently demonstrated the greatest delaying effects on the phases of Evening locomotor peaks; they were also among the sites with greatest influence on the amplitudes of the Morning and Evening peaks. The results suggest their phosphorylation normally will have the greatest effect to slow or terminate PDFR signaling. Other sites are also effective, although to lesser degrees (as indicated by their font sizes), including CL2-3, CL4 and CL5. Primarily, such phosphorylation will decrease the duration of PDFR signaling (red bars) and so reduce (for example) the delay that PDF imposes on the period of neuronal activation displayed by PDFR-responsive pacemaker groups (like the Evening cells and the DN3 [10, 19]). The model also predicts that the effects of phosphorylating CL5 will depend on the phosphorylation status of neighboring sites. In some contexts (e.g., PDFR 1-5A), it will help terminate PDFR signaling, but in others (e.g., PDFR 567A) it may promote the duration or extent of PDFR signaling, perhaps by blocking the effects of phosphorylating CL6 and CL7 (green bar).

The 1-7A PDFR variant modified the greatest number of phosphorylatable residues in the series we tested (**Fig 1**), and typically produced the strongest behavioral effects. Notably, the 6A variant–which modified only two specific Ser residues–produced effects that were nearly identical to those exhibited by flies expressing the 1-7A form. This suggests that much PDFR post-translational modification, that which is capable of restricting its signaling time-course, may be directed to specific phosphorylation sites near the extreme C terminal end. There are two broadly divergent hypotheses to describe the mechanisms by which GPCR phosphorylation promotes desensitization. The first proposes that modification of specific residues have the greatest significance for downstream desensitizing mechanisms: such a mechanism can explain the effects we have seen with the highly limited 6A PDFR variant. The second hypothesis invokes triggering of a termination processes by the aggregate negative charge accumulated with bulk phosphorylation, regardless of where it might occur along a GPCR’s intracellular sequences. For some GPCRs (e.g., OPN4), the evidence supports both models [34,53]. Our data concerning PDFR desensitization also support both viewpoints and we present a model in **Fig 7**. In particular we regard CL6 and CL7 to be the specific residues especially critical in terminating the time course of PDFR signaling. Notably, converting just the single pair of CL6 AAs, or the single pair of CL7 AAs, is enough to generate hours-long delays in the peaks of locomotor rhythms under Light:Dark conditions. We note that the splicing event that distinguishes the PDFR-A and PDFR-D isoforms occurs just prior to the position of sequences encoding CL6 and 7, such that the D form lacks these two highly conserved domains (flybase.org/download/sequence/FBgn0260753/FBpp). Other clusters (like CL2-3) also appear to have potential for ‘specific’ contributions to PDFR regulation. Together, these results speak to the potency of the inferred specific termination mechanisms for PDFR GPCR signaling. In contrast, the evidence for the bulk phosphorylation hypothesis comes from comparison of the 1-4A, versus the 1-5A, 1-6A and 1-7A variants. This series inactivates increasing numbers of phosphorylatable residues, and with it we observed increasingly delayed evening phases in short day conditions (e.g., **Fig 2I–2 though 2L-2**). The tandem mass-spectroscopy results, indicating endogenous phosphorylation of certain PDFR residues (**S4 Table**) demonstrates such post-translational modifications can occur. However, we caution that these phospho-peptide measurements derive from whole head extracts and may not provide a complete or relevant accounting of those sites that are modified in critical E pacemaker neurons.

Our results also point to what we propose as “context-dependent” effects of modifying specific GPCR residues–ones that may produce opposing behavioral effects, depending on the phosphorylation status of neighboring sites. In particular we point to the opposing results we observed with Ala-variants of the CL5 site. In comparing results from the series 1-4A, 1-5A, 1-6A and 1-7A, we found that under Short Day conditions, the 1-4A had only mild effects on the evening peak phase compared to over-expressing WT PDFR (e.g., Fig **2B–2** vs. **2I-2**). Whereas 1-5A, 1-6A and 1-7A all produced significant delays (**Figs 2M and 3I-3 through 3L-3)**. The difference between the 1-$A and 1-5A variants is a change in a single S residue at CL5 (S633A): Those observations suggest modification of the CL5 residue normally promotes desensitization of PDFR and termination of PDFR signaling. However, the PDFR variant 5-7A was constructed with the expectation that it would be as effective as 1-5A, 1-6A or 1-7A in delaying the evening phase. Instead it proved only weakly effective. This same mis-match of expected behavioral effects for 5-7A was also seen in other photoperiodic conditions (**Fig 6N and 7H-3 vs 7J-3 through 7L-3).** Likewise, Langlet *et al*. [36] reported that effects on mutating Serine residues in the carboxy terminus of VPAC1 did not produce additive effects. We propose that phosphorylation of CL5 will have either positive or negative consequences on PDFR desensitization depending on which other neighboring residues are also modified. Thus we speculate that CL5 takes on outsize importance in determining desensitization rates for PDFR and so may itself be subject to exceptional regulation. Such complex interactions between phosphorylation sites is reminiscent of interactions documented between diverse phosphorylation sites in the circadian clock protein PERIOD [54–55]. Better resolution of these two paradoxical mechanisms ((i) specific phosphorylation versus bulk negative charge, and (ii) context-dependent effects of CL5 site phosphorylation, awaits precise molecular definition of where and when PDFR is modified in key pacemaker neurons *in vivo*, and by which post-translational modifications.

### Validating the behavioral phenotypes produced by PDFR variants

The actions of PDFR variants we have described, following substitutions of Ala for various Ser/Thr or Tyr residues, are not easily explained by a hypothesis invoking neomorphic or constitutive GPCR properties. The evidence for this conclusion is three-fold. First, the effects on both amplitude and phase of activity peaks by these variants are not of a random assortment: rather they are all strictly opposite to that of loss-of-function models for *pdf* and *pdfr*. Second, the actions of PDFR variants display strong dependence on wild type *pdf* gene function: this strongly argues that the actions of PDFR variants reflect responses to normal, endogenous PDF signaling. Third, even the strongest actions of PDFR variants on the phases and amplitudes of locomotor activity (that of the 1-7A variant) reflected PDFR sequence variation, and not the properties of the GFP C-terminal fusion. Together these observations support the hypothesis that phosphorylation of some or all of the C terminal residues we studied are normally modified to attenuate the strength and duration of PDFR signaling during the 24-hr day.

### PDF Signaling and seasonal adaptation

PDF neuropeptide actions were discovered in the context of physiological adaptions to daylight in crustacea [5,6]. Helfrich-Förster [56] discovered PDF expression within a defined subset of the insect *Drosophila* circadian pacemaker network. Renn *et al*. [1] demonstrated that *pdf* makes a fundamental contribution by setting the normal behavioral phase of Evening activity in *Drosophila* during light:dark entrainment and promoting normal rhythmicity during constant darkness. PDF signaling coordinates with that driven by light: It works in parallel to direct photosensitivity along with the CRY blue light photoreceptor. Flies doubly-mutant for *cry* and *pdf* display pronounced deficits of locomotor rhythmicity [7, 8, 9], and can be as severe as measured in clock-deficient flies. PDF and environmental light also work coordinately at the level of neuronal activity patterns: in the case of the evening pacemakers (LNd and the 5^th^ s-LNv), both light and PDF signaling promote a delay in their PER-dependent activation period, and together help align it to a phase just prior to dusk [19]. Based on the close association of PDF signaling and photoperiodic signaling, it is not surprising that the duration of the photoperiod strongly influenced the effects of PDFR sequence variants on the Morning versus the Evening activity peaks. We only observed delayed Evening activity peak produced by PDFR gain-of-function variants under Short Day conditions: that delay appears strongly inhibited by light durations > 8 hr. It remains unclear at what molecular level light exerts such an inhibitory effect, however, we note a similarity of these observations with effects recently reported from loss-of-function states for the phosphatase *PRL-1* [57]. Like the most active PDFR variants we herein describe (e.g., 6A, 7A, 1-5A 1-6A and 1-7A), *PRL-1* mutants produce a 3–4 h delay in the phase of the evening activity peak, but only under winter-like (short day) conditions, and not under equinox or summer-like (long day) conditions. Kula-Eversole *et al*. [57] have shown that TIM phosphorylation is affected by PRL-1 activity and suggest the seasonal action of PRL-1 to advance the evening locomotor activity phase is mediated by modification of TIM levels within the transcription-translation feedback loop. The extensive similarity of these two sets of behavioral phenotypes reveals either serial or parallel pathways to effect comparable outcomes on locomotor activity. If serial, then according to the simplest model, PRL1 acts downstream of PDFR, and the combined results to-date suggest PRL-1 would be inhibited by PDFR activation. Further genetic and biochemical experiments are needed to evaluate if and how these pathways converge.

### Relations between in vitro and in vivo PDFR signaling measures

Using an *in vitro* assay for cyclic AMP generation, we found that modifying phosphorylation properties of PDFR does not affect the strength of signaling. That conclusion suggests that *in vivo* other GPCR features are normally affected to regulate the extent/duration of PDFR signaling according to season. This speculation corresponds to findings described with other Family B GPCRs like VPAC1 [47]: Mutation of all the Ser and Thr residues of the C terminal tail, and of Ser250 to Ala, led to a receptor with binding properties and adenylate cyclase activity similar to the wild type receptor; however that variant receptor was neither phosphorylated nor internalized. We propose that the PDFR variants we have described do not modify locomotor behavior by virtue of greater second messenger signaling. Rather they do so by generating cAMP (and perhaps other second messengers) over time periods longer than that normal sustained by a WT receptor over a portion of each 24-hr cycle.

### Is PDFR modulation of diurnal phase independent of its modulation of circadian period?

A conventional interpretation of the phenotypes we have described is that PDFR signaling affects the pace of the molecular clock (re-setting the period) and consequently affects behavioral phase indirectly, which is downstream of the clock. However, PDFR variants reliably delayed the Evening activity phase under winter-like conditions, but these variants did not significantly lengthen circadian period. All effects of the 1-7A variant on circadian period were derived from the GFP fusion. Therefore, we cannot rule out an alternative hypothesis: that the duration of PDF>PDFR signaling to target pacemakers in the *Drosophila* brain is modulated each day to directly delay Evening pacemaker neuronal activity independent of its effects on entrainment of the molecular clock. In fact, the *pdfr han* mutant flies exhibited quasi-normal tau values in this report (Table 1), although they more traditionally exhibit shortened ones in our experience [38]. Evening locomotor activity derives in large part from the activity of the primary Evening oscillators, termed E cells, the LNd and the 5^th^ small LNv [10,20,58–60]. However, numerous network interactions are also known to provide critical contributions to the accuracy, precision and adaptability of that timing system [e.g., 61–65]. The Evening oscillator group itself is known to be heterogeneous and to constitute at least three separate, functionally distinct oscillators [62], only some of which express PDFR-A and respond to PDF [38,62]. Vaze and Helfrich-Förster [66] reported that the phase of Period protein accumulation in E neurons is phase-locked to the previous lights-off transition and also sensitive to PDF signaling. They conclude that the peak of PER accumulation is key to determining the phase of the evening activity peak, but also note that the correlation between the Period-clock timing cue and the Evening activity peak is not perfect. They suggest the Evening behavioral phase is better described by also factoring in the delay in E cell neuronal activity driven by PDF neuromodulation [10, 19]. We speculate that it is the combination of two separate PDFR signaling effects that is essential for proper rhythmic behavioral outcomes across seasons. If valid, the hypothesis predicts a bifurcation of signaling pathways downstream of PDFR, to independently regulate diurnal phase and also circadian period within E pacemaker neurons.

## Materials and methods

### Fly rearing and stock

*Drosophila* were raised on a cornmeal agar diet supplemented with yeast at 25°C in 12 hr:12 hr LD cycles. The UAS-*pdfr* mutant transgenic series was created by injecting *yw P{nos phiC31\int*.*NLS}X;P{CaryP}attP40* embryos (Rainbow Transgenic Flies, Inc, Camarillo, CA). The UAS-*pdfr*-tandem construct was injected into y[1] w[*] P{y[+t7.7] = nos-phiC31\int.NLS}X; P{y[+t7.7] = CaryIP}su(Hw)attP6 embryos. For PDFR-tandem fusion protein expression, we made a stable *yw*; *tim*(UAS)-Gal4; UAS-*pdfr*-tandem stock. *pdfr* mutant flies are described in [11]; *tim*(UAS)-Gal4 flies are described in [67]–*BL80941*). The recombinant fly stock—*han*^*5304*^; *tim* (UAS)-Gal4—was confirmed by PCR and sequencing.

### *hEK-293* cell culture

*hEK-293* cells were maintained in DMEM, 10% FBS and 100U/mL penicillin and streptomycin in 5% CO_2_ atmosphere at 37°C. For all transient transfections, 1.5 x 10^6^ cells were used to inoculate T25 flasks, incubated overnight, then transfected with 10ug plasmid DNA and 20 uL lipofectamine 2000 reagent (Invitrogen Life Technologies). Five hours after transfection, cells were split 4 x 10^4^ cells/well into a 96-well assay plate. We created a series of stably-transfected *hEK-293* cells expressing WT and sequence-variant PDFRs, using the Flp-In System (Invitrogen Life Technologies, Waltham, Massachusetts) per manufacturers recommendations, and maintained them in DMEM supplemented with 150 μg/ml hygromycin B.

### cAMP assays

We measured PDF Receptor signaling activity using a CRE-*Luciferase* reporter gene, following methods described by **Johnson et al.** [30]. The reporter gene construct was transiently transfected to each stable cell line and luminescence measured using Firefly Luciferase Assay Kit (Biotium, Inc., Fremont, California) and a Wallac 1420 VICTOR2 microplate reader (PerkinElmer, Inc., Waltham, Massachusetts). Concentration-effect curves, EC_50_, top values and p-values were calculated using the dose response, in a nonlinear regression using GraphPad Prism 8.0 software (San Diego, California).

### Locomotor activity measures

All locomotor activity experiments were conducted with 2–5 days-old male flies at 25°C using Trikinetics Activity Monitors as previously described [9]. We crossed Gal4 lines to *w*^*1118*^ to create control progeny. Locomotor activities were monitored for 6 days under different photoperiodic conditions, and then for 9 days under constant dark (DD). To analyze rhythmicity under constant conditions, we normalized the activity of flies from DD day 3 to day 9 and used χ2-periodogram analysis with a 95% confidence cut-off, as well as SNR analysis [68]. Arrhythmic flies were defined by a power value ≤ 10 and width value ≤ 2, and period outside the range, 18 to 30 hours. To analyze periods, we used Graphpad Instat (v. 8) software to run one-way ANOVA measures followed by the Tukey-Kramer Multiple Comparisons Test. We used Clocklab (Actimetrics) software to produce actograms and the Brandeis Rhythms Package [69] to produce average activity plots (group eductions). To analyze onset and offset phases of Morning and Evening activity bouts on the final two days of light:dark entrainment (LD 5&6), we followed the method of Kula-Eversole *et al*. [57]: [(A_n+2_ + A_n+1_)–(A_n-1_ + A_n-2_)  =  ΔActivity]. Experimental genotypes were tested in *the han* mutant background, *pdfr*^*5304*^ *[11], o*r in the *pdf*^*01*^ (null) mutant background [1], or in the *w*^*1118*^ background, as noted.

### Presentation of behavioral data with expression of PDFR variants

Figs 2–4 all describe rhythmic locomotor behavior of adult *pdfr*^*han*^ mutant flies that are expressing either a WT *pdfr* cDNA or the 1-7A variant. Figs 5 and 6 use a similar format but they introduce behavioral experiments performed in *pdfr*^*+*^ or *pdf* mutant backgrounds, as described. All behavioral experiments extend across ~6 days of light entrainment (LD), followed by activity during nine or more days of constant darkness (DD). For each genotype, we display locomotor activity during light entrainment in four ways: (i) group eductions (in panels marked **A, B and C**-**1** in these Figures), (ii) direct comparisons between two genotypes of the amplitudes of behavioral peaks using bin-by-bin analyses during the last two days of entrainment (days 5–6) (in panels marked **A, B and C-2-4**); (iii) derivations of Morning and Evening behavioral phases (in Panels **D and E**); and (iv) double-plotted group actograms (in Panels **F, G and H**). The bin-by-bin analyses directly compare experimental genotypes to a control one [cf. 70]. They present data averaged across 2 to 5 independent experiments, to help identify the differences that most consistently correlate with genotype. Finally, Table 1 compiles measures of rhythmic activity displayed by the different genotypes in days 3–9 of constant darkness.

Additional details on Methods and procedures are found in the text file S1 Text.

## Supporting information

S1 Table Accession numbers for PDFR-A from 17 *Drosophalid* species used to assess evolutionary conservation of individual AA residues in the C terminal regions. (PDF)

S2 Table Oligonucleotides used in this study for cloning. (PDF)

S3 Table Potential phosphorylatable residues in the C terminal of the *D*. *melanogaster* PDFR-A isoform. (PDF)

S4 Table Phosphopeptides derived from PDFR-Tandem expression, detected *in vivo*. (PDF)

S5 Table Rhythmic behavior under Constant Dark Conditions for flies in which *GRK1*, *GRK2* and *β-arrestin-2* are manipulated. (PDF)

S1 Fig Alignment of the C terminal PDFR-A sequences from 17 different *Drosophalid* species. The predicted 7^th^ transmembrane domain (TM7) is marked in GREY. The C terminal tail starts with V505 (numbering for the *melanogaster* protein). The 28 potentially phosphorylated residues in the D.m. C terminal tail are highlighted in color: the 14 residues chosen for analysis are marked by their Cluster (CL) designation (1 to 7) and marked in AQUA; the 14 non-selected residues are marked in YELLOW. See **S2 Table** for additional sequence information.
(PDF)

S2 Fig Locomotor Rhythms exhibited by WT PDFR and by other PDFR Variants under Short-day (winter-like) condition. All behavioral records were recorded from *han* (*pdfr* mutant) flies that expressed either no UAS transgene (A), or a UAS-WT *pdfr* transgene (B) or a variety of Simple *pdfr* Variants, including 2-3A (C), 4A (D), 5A (E), 6A (F) or 7A (G), or Multiple *pdfr* variants, including 5-7A (H), 1-4A (I), 1-5A (J), and 1-6A (K). Top Right Box: A schematic of the PDFR C terminal segment for the WT and all variants studied: see Figure Legend 1 for details. Letters to the right of each variant C terminal segment correspond to the Panels in this Figure that display the behavior observed following its expression. Each Panel (A)-(K) contains sub-panels (1) and (2): Sub-Panel (1) displays a daily plot of locomotor activity (a group eduction) averaged over the last two days of entrainment (LD 5–6). Open bars indicate the 8 hr periods of Lights-on and filled bars indicate 16 hr periods of Lights-off. Sub-panel (2) displays a double-plotted group actogram throughout the 6 days of LD entrainment, followed by ~9 days of (DD, grey background). Green lines indicate the phase of the dominant activity period in DD. Panel L displays the average Morning activity Phase Onset timepoint, and Panel M displays the average Evening activity Phase Offset (marked by a Blue Arrow) for each genotype over the last two days of entrainment (LD 5–6). The positions of the Red and Blue arrows in panels A-K are representative phase points; panels L and M present their true values respectively. Analyses represent ANOVA followed by Dunnett’s post hoc multiple comparisons of all compared to WT: ns = not significant; * = p<0.05; ** = p<0.01; *** = p<0.005; **** = p<0.001.
(PDF)

S3 Fig Amplitude measures of locomotor rhythms exhibited by WT PDFR and by other PDFR Variants under Short-day (winter-like) conditions. All behavioral records were recorded from *han* (*pdfr* mutant) flies that expressed either no UAS transgene (A–marked in YELLOW), or a WT *pdfr* cdNA (B–marked in BLACK) or a variety of Simple *pdfr* Variants (all marked in RED) including 2-3A (C), 4A (D), 5A (E), 6A (F), 7A (G), 5-7A (H), 1-4A (I), 1-5A (J), and 1-6A (K). These measures were averaged across all experiments run for each individual genotype (see Table 1 for N and n values). Top Right Box: A schematic of the PDFR C terminal segment for the WT and all variants studied: see Figure Legend 1 for details. Letters to the right of each variant C terminal segment correspond to the Panels in this Figure that display the behavior observed following its expression. Each Panel (A)-(L) contains sub-panels (1) through (3), each of which displays Bin-by-Bin analyses of activity levels sorted by 30 min bins, for three different time periods: (1) ZT17-23.5; (2) ZT 0.5–8; (3) ZT 8.5–16. The missing bin at timepoint 0 contains the startle response that accompanies the sudden lights-on signal. Blue asterisks indicate significantly-different activity levels according to a Student’s T-test following an ANOVA (p< 0.05). Red arrows highlight elevated Morning activity levels displayed by different PDFR variants. Blue arrows highlight elevated Evening activity levels produced by different PDFR variants.
(PDF)

S4 Fig Locomotor Rhythms exhibited by WT PDFR and by other PDFR Variants under Equinox (12:12) condition. All behavioral records were recorded from *han* (*pdfr* mutant) flies that expressed either no UAS transgene (A), or a UAS-WT *pdfr* transgene (B) or a variety of Simple *pdfr* Variants, including 2-3A (C), 4A (D), 5A (E), 6A (F) or 7A (G), or Multiple *pdfr* variants, including 5-7A (H), 1-4A (I), 1-5A (J), and 1-6A (K). Top Right Box: A schematic of the PDFR C terminal segment for the WT and all variants studied: see Figure Legend 1 for details. Letters to the right of each variant C terminal segment correspond to the Panels in this Figure that display the behavior observed following its expression. Each Panel (A)-(L) contains sub-panels (1) and (2): Sub-Panel (1) displays a daily plot of locomotor activity (a group eduction) averaged over the last two days of entrainment (LD 5–6). Open bars indicate the 12 hr periods of Lights-on and filled bars indicate 12 hr periods of Lights-off. Sub-panel (2) displays a double-plotted group actogram throughout the 6 days of LD entrainment, followed by ~9 days of (DD, grey background). Green lines indicate the phase of the dominant activity period in DD. Panel L displays the average Morning activity Phase Onset timepoint, and Panel M displays the average Evening activity Phase Onset (marked by a Blue Arrow) for each genotype over the last two days of entrainment (LD 5–6). The positions of the Red and Blue arrows in panels A-K are representative phase points; panels L and M present their true values respectively. Analyses represent ANOVA followed by Dunnett’s post hoc multiple comparisons of all compared to WT: ns = not significant; * = p<0.05; ** = p<0.01; *** = p<0.005; **** = p<0.001.
(PDF)

S5 Fig Amplitude measures of locomotor rhythms exhibited by WT PDFR and by other PDFR Variants under Equinox (12:12) conditions. All behavioral records were recorded from *han* (*pdfr* mutant) flies that expressed either no UAS transgene (A–marked in YELLOW), or a WT *pdfr* cdNA (B–marked in BLACK) or a variety of Simple *pdfr* Variants (all marked in RED) including 2-3A (C), 4A (D), 5A (E), 6A (F), 7A (G), 5-7A (H), 1-4A (I), 1-5A (J), and 1-6A (K). These measures were averaged across all experiments run for each individual genotype (see Table 1 for N and n values). Top Right Box: A schematic of the PDFR C terminal segment for the WT and all variants studied: see Figure Legend 1 for details. Letters to the right of each variant C terminal segment correspond to the Panels in this Figure that display the behavior observed following its expression. Each Panel (A)-(L) contains sub-panels (1) through (3), each of which displays Bin-by-Bin analyses of activity levels sorted by 30 min bins, for three different time periods: (1) ZT17-23.5; (2) ZT 0.5–8; (3) ZT 8.5–16. The missing bin at timepoint 0 contains the startle response that accompanies the sudden lights-on signal. Blue asterisks indicate significantly-different activity levels according to a Student’s T-test following an ANOVA (p< 0.05). Red arrows highlight elevated Morning activity levels displayed by different PDFR variants. Blue arrows highlight elevated Evening activity levels produced by different PDFR variants.
(PDF)

S6 Fig Locomotor Rhythms exhibited by WT PDFR and by other PDFR Variants under Long-day (summer-like) condition. All behavioral records were recorded from *han* (*pdfr* mutant) flies that expressed either no UAS transgene (A), or a UAS-WT *pdfr* transgene (B) or a variety of Simple *pdfr* Variants, including 2-3A (C), 4A (D), 5A (E), 6A (F) or 7A (G), or Multiple *pdfr* variants, including 5-7A (H), 1-4A (I), 1-5A (J), and 1-6A (K). Top Right Box: A schematic of the PDFR C terminal segment for the WT and all variants studied: see Figure Legend 1 for details. Letters to the right of each variant C terminal segment correspond to the Panels in this Figure that display the behavior observed following its expression. Each Panel (A)-(L) contains sub-panels (1) and (2): Sub-Panel (1) displays a daily plot of locomotor activity (a group eduction) averaged over the last two days of entrainment (LD 5–6). Open bars indicate the 16 hr periods of Lights-on and filled bars indicate 8 hr periods of Lights-off. Sub-panel (2) displays a double-plotted group actogram throughout the 6 days of LD entrainment, followed by ~9 days of (DD, grey background). Green lines indicate the phase of the dominant activity period in DD. Panel L displays the average Morning activity Phase Offset timepoint, and Panel M displays the average Evening activity Phase Onset (marked by a Blue Arrow) for each genotype over the last two days of entrainment (LD 5–6). The positions of the Red and Blue arrow in panels A-K are representative phase points; panels L and M present their true values respectively. Analyses represent ANOVA followed by Dunnett’s post hoc multiple comparisons of all compared to WT: ns = not significant; * = p<0.05; ** = p<0.01; *** = p<0.005; **** = p<0.001.
(PDF)

S7 Fig Amplitude measures of locomotor rhythms exhibited by WT PDFR and by other PDFR Variants under Long-day (summer-like) conditions. Behavioral records recorded from *han* (*pdfr* mutant) flies that expressed either no UAS transgene (A–marked in YELLOW), or a WT *pdfr* cdNA (B–marked in BLACK) or a variety of Simple *pdfr* Variants (all marked in RED) including 2-3A (C), 4A (D), 5A (E), 6A (F), 7A (G), 5-7A (H), 1-4A (I), 1-5A (J), and 1-6A (K). These measures were averaged across all experiments run for each individual genotype (see Table 1 for N and n values). Top Right Box: A schematic of the PDFR C terminal segment for the WT and all variants studied: see Figure Legend 1 for details. Letters to the right of each variant C terminal segment correspond to the Panels in this Figure that display the behavior observed following its expression. Each Panel (A)-(L) contains sub-panels (1) through (3), each of which displays Bin-by-Bin analyses of activity levels sorted by 30 min bins, for three different time periods: (1) ZT17-23.5; (2) ZT 0.5–8; (3) ZT 8.5–16. The missing bin at timepoint 0 contains the startle response that accompanies the sudden lights-on signal. Blue asterisks indicate significantly-different activity levels according to a Student’s T-test following an ANOVA (p< 0.05). Red arrows highlight elevated Morning activity levels displayed by different PDFR variants. Blue arrows highlight elevated Evening activity levels produced by different PDFR variants.
(PDF)

S8 Fig Average daily locomotor rhythms in flies expressing WT and 1-7A variant PDFR transgenes that lack GFP fusions. All behavioral records were recorded from *han* (*pdfr* mutant) flies that expressed either no UAS transgene (panels (A, D, I, and L)—yellow), or a WT *pdfr* cdNA (panels (B, E, J and M)—black) or the PDFR 1-7A Multiple Variant (panels (C, F, K and N)–red). Panels (A-F) present data recorded under Short-Day (winter-like) conditions; panels (I-N) present data recorded under Long Day (summer-like) conditions. Each Panel (A—C) and (I -K) contains four sub-panels: Sub-Panel (1) displays an average daily plot of locomotor activity averaged over the final two days of light entrainment (a group eduction): Open bars indicate the periods of Lights-on and filled bars indicate periods of Lights-off. Sub-Panels 2–4 display Bin-by-Bin analyses of activity levels sorted by 30 min bins, for three different time periods: (1) ZT17-23.5; (2) ZT 0.5–8; (3) ZT 8.5–16. The missing bin at timepoint 0 contains the startle response that accompanies the sudden lights-on signal. Blue asterisks indicate significantly-different activity levels according to a Student’s T-test following an ANOVA (p< 0.05). Panels (D—F) and (L—N) display double-plotted group actograms throughout the 6 days of Light: dark entrainment, followed by 9 days of constant darkness (DD, grey background). Panels (G) and (H) display the average Phase Onsets and Offsets (respectively) for the Morning and Evening activity periods for each genotype over the last two days of entrainment under short days (LD 5–6). Blue arrows highlight the elevated Evening activity amplitudes. Panels (O) and (P) display the average Phase Offsets and Onsets (respectively) for the Morning and Evening activity periods for each genotype over the last two days of entrainment under long days (LD 5–6). Red Arrows highlight the elevated amplitude of the Morning peak. Ns–not significant; *—p < 0.05; ***—p < 0.005; ****—p < 0.001.
(PDF)

S9 Fig Basal cAMP signaling displayed by the PDFR variant series following functional expression *in vitro*. Luciferase measurements in *hEK-293T* cells stably expressing WT PDFR or its variants and transiently expressing *CRE-Luciferase*. The histogram represents basal levels of 2^nd^ messenger signaling, i.e., in the absence of stimulation by neuropeptide PDF. Values represent the mean +/-SEM of three independent measurements, and were analyzed by Student ‘s T-test: * = p < 0.05; ns = not significantly different.
(PDF)

S10 Fig EC50 values for cAMP generation displayed by the PDFR variant series following functional expression *in vitro*. Luciferase measurements in *hEK-293T* cells stably expressing WT PDFR or its variants and transiently expressing *CRE-Luciferase*. The histograms represent EC50 values for PDF-stimulated 2^nd^ messenger signaling. Values represent the mean +/-SEM of three independent measurements, and were analyzed by Student ‘s T-test: * = p < 0.05; ns = not significantly different.
(PDF)

S11 Fig Top Values for cAMP generation displayed by the PDFR variant series following functional expression *in vitro*. Luciferase measurements in *hEK-293T* cells stably expressing WT PDFR or its variants and transiently expressing *CRE-Luciferase*. The histograms represent the top values achieved for 2^nd^ messenger signaling following PDF-stimulation. Values represent the mean +/-SEM of three independent measurements, and were analyzed by Student ‘s T-test: * = p < 0.05; ns = not significantly different.
(PDF)

S12 Fig Surface expression of the PDFR variant series following functional expression *in vitro*. β -Lactamase activity measurements in *hEK-293T* cells stably expressing WT PDFR or its variants fused to β -lactamase at the N terminus. The histograms represent the basal values for surface receptor expression in the absence of stimulation by neuropeptide PDF. Values represent the mean +/-SEM of three independent measurements, and were analyzed by Student ‘s T-test: * = p < 0.05; ns = not significantly different.
(PDF)

S13 Fig Percentage change in surface expression of the PDFR variant series after exposure to PDF, following functional expression *in vitro*. β-Lactamase activity measurements in *hEK-293T* cells stably expressing WT PDFR or its variants fused to β -lactamase at the N terminus. The histograms represent the values for surface receptor expression 20 m after exposure to neuropeptide PDF. Values represent the mean +/-SEM of three independent measurements, and were analyzed by Student ‘s T-test: * = p < 0.05; ns = not significantly different.
(PDF)

S14 Fig *In vivo* detection of phosphopeptides from the PDFR C terminal tail. Nine spectra of phosphopeptides derived from a UAS-*PDFR*-Tandem construct from eight independent immunoprecipitation experiments.
(PDF)

S15 Fig Average daily locomotor rhythms in flies with manipulations of *GRK1*, *GRK2* and *β-arrestin2*. Group eductions of locomotor activity profiles for different genotypes averaged over six days of light entrainment. Open bars indicate periods of Lights-On and filled bars indicate periods of Lights-Off. The column present manipulations for each of four different gene targets (none; *Gprk1*; *Gprk2* and *β-arr2* ***(****kurtz))* using *tim*(UAS)-Gal4. The Upper panels (D and F) present over-expression experiments. The Middle (E and G) and Lower panels (H and I) present RNAi experiments. UAS-RNAi constructs illustrated in panels E and G were created and shared by the Paul Hardin laboratory. Values for N and n are found in S5 Table.
(PDF)

S1 Text Additional details on methods and procedures. (DOCX)
