# Supplementary material for: Regulation of PDF receptor signaling controlling daily locomotor rhythms in Drosophila
Source: PLoS Genet. 2022 May 23;18(5):e1010013. doi: 10.1371/journal.pgen.1010013 (PMC9166358; doi:10.1371/journal.pgen.1010013)
Supplement: S1 Table — (PDF) [file pgen.1010013.s001.pdf]

S1 Table. *Drosophalid* PDFR Accession Numbers

Genus/ Species	Accession Number	Total AA length
Drosophila melanogaster	NP_570007.2	669
Drosophila takahashii	XP_016994843.1	693
Drosophila ficusphila	XP_017048318.1	679
Drosophila rhopaloa	XP_016982487.1	671
Drosophila eugracilis	XP_017067611.1	675
Drosophila erecta	EDV45631.1	671
Drosophila simulans	KMZ07909.1	717
Drosophila persimilis	EDW26013.1	591
Drosophila miranda	XP_033242917.1	685
Drosophila willistoni	EDW82885.2	663
Drosophila bipectinate	XP_017095130.1	684
Drosophila albomicans	XP_034119693.1	702
Drosophila grimshawi	EDW00227.1	679
Drosophila virilis	XP_032288784.1	623
Drosophila hydei	XP_030080101.1	668
Drosophila novamexicana	XP_030566778.1	623
Drosophila navajoa	XP_017964591.1	671

S1 Table. Accession numbers for PDFR-A from 17 Drosophalid species used to assess evolutionary conservation of individual AA residues in the C terminal regions.
