# Supplementary material for: Regulation of PDF receptor signaling controlling daily locomotor rhythms in Drosophila
Source: PLoS Genet. 2022 May 23;18(5):e1010013. doi: 10.1371/journal.pgen.1010013 (PMC9166358; doi:10.1371/journal.pgen.1010013)
Supplement: S2 Table — (PDF) [file pgen.1010013.s002.pdf]

S2 Table.
Oligonucleotides used for cloning

β-Lac 13758 pcDNA3 vector construction : digest 13758 PCR & β-Lac GBR1 pcDNA3 with AscI & Not1 and ligate.

c13758 AscI-F	CCCGGCGCGCCCaccctcctgtcgaacattctcgactgcggaggc
c13758 Not1-R	CCCGCGGCCGCctactgctctgacaactcaaatacaactgactc

β-Lac pcDNA5 vector construction: digest β-Lac PCR & pcDNA5 with Nhe1 & HindIII and ligate

Blac atg Nhe-F	ggGCTAGCatggctagtgagacagacacactcctgcta
Blac end HindIII-R	ggAAGCTTccaatgcttaatcagtgaggcacctatctcag

β-Lac 13758 WT pcDNA5 vector construction: Digest 13758 PCR & β-Lac pcDNA5 with HindIII & Not1 and ligate.

c13758 HindIII-F	ggaagcttGCCACCATGACCCTCCTGTCGAACATTCTC
c13758 Not1-R	CCCGCGGCCGCctactgctctgacaactcaaatacaactgactc

β-Lac 13758 SNA pcDNA3 C-terminal mutagenesis:

c13758 AscI-F	CCCGGCGCGCCCaccctcctgtcgaacattctcgactgcggaggc
c13758 S1A-R	gacctcgcacTGCcagctgggtggccagTGCctttagtag
c13758 S2A-R	cgtatccggcgccgtgttTGCagcaccTGCTGCcatTGCtgccctttttcggcgccca
c13758 S3A-R	tgcaggctgcactgcatcTGCatccggcgcTGCgttataagcacccgag
c13758 S4A-R	gcggtgatattcgctttccTGCggccgatggatctcctgc
c13758 S5A-R	cgcgctgcccgcctcctttTGCgtgaatgtgggagatggtc
c13758 S6A-R	caactgactcgggtggcacTGCTGCcgctgacggtactcttag
c13758 S7A-R	ggttttggtctactgctcTGCcaactcaaatacaacTGCctcgggtggcacggatgac

β-Lac S23A 13758 pcDNA3 C-terminal mutagenesis:

Performed mutagenesis on β-Lac S2A 13758 pcDNA3 construct:

c13758 AscI-F	CCCGGCGCGCCCaccctcctgtcgaacattctcgactgcggaggc
c13758 S3A-R	tgcaggctgcactgcatcTGCatccggcgcTGCgttataagcacccgag

β-Lac S567A 13758 pcDNA3 C-terminal mutagenesis:

Performed mutagenesis on β-Lac S5A 13758 pcDNA3 construct:

c13758 AscI-F	CCCGGCGCGCCCaccctcctgtcgaacattctcgactgcggaggc
c13758 S6A-R	caactgactcgggtggcacTGCTGCcgctgacggtactcttag

followed by mutagenesis with B-Lac S56A 13758 pcDNA3 construct:

c13758 AscI-F	CCCGGCGCGCCCaccctcctgtcgaacattctcgactgcggaggc
c13758 S7A-R	ggttttggtctactgctcTGCcaactcaaatacaacTGCctcgggtggcacggatgac

β-Lac S1-4A 13758 pcDNA3 C-terminal mutagenesis:

Performed mutagenesis on β-Lac S1A 13758 pcDNA3 construct:

c13758 AscI-F	CCCGGCGCGCCCaccctcctgtcgaacattctcgactgcggaggc
c13758 S2A-R	cgtatccggcgccgtgttTGCagcaccTGCTGCcatTGCtgccctttttcggcgccca

followed by mutagenesis with β-Lac S12A 13758 pcDNA3 construct:

c13758 AscI-F	CCCGGCGCGCCCaccctcctgtcgaacattctcgactgcggaggc
c13758 S3A-R	tgcaggctgcactgcatcTGCatccggcgcTGCgttataagcacccgag

followed by mutagenesis with β-Lac S123A 13758 pcDNA3 construct:

c13758 AscI-F	CCCGGCGCGCCCaccctcctgtcgaacattctcgactgcggaggc
c13758 S4A-R	gcggtgatattcgctttccTGCggccgatggatctcctgc

β-Lac S1-7A 13758 pcDNA3 C-terminal mutagenesis:

Performed mutagenesis on β-Lac S1-4A 13758 pcDNA3 construct:

c13758 AscI-F	CCCGGCGCGCCCaccctcctgtcgaacattctcgactgcggaggc
c13758 S5A-R	cgcgctgcccgcctcctttTGCgtgaatgtgggagatggtc

followed by mutagenesis with β-Lac S1-5A 13758 pcDNA3 construct:

c13758 AscI-F	CCCGGCGCGCCCaccctcctgtcgaacattctcgactgcggaggc
