# Supplementary material for: Regulation of PDF receptor signaling controlling daily locomotor rhythms in Drosophila
Source: PLoS Genet. 2022 May 23;18(5):e1010013. doi: 10.1371/journal.pgen.1010013 (PMC9166358; doi:10.1371/journal.pgen.1010013)
Supplement: S3 Table — (PDF) [file pgen.1010013.s003.pdf]

S3 Table. Potential phosphorylatable residues in the C terminal of the PDFR-A isoform

Residue	conservation among 17 species	Cluster designation	phosphorylated *in vivo?*
S512	100%	CL1	
T514	53%		
S518	100%	CL1	
S531	100%	CL2	YES
Y533	100%	CL2	
S534	100%	CL2	YES
Y537	100%	CL2	
T539	100%	CL3	
T543	100%	CL3	
S554	35%		
T556	100%	CL4	
S560	94%		YES
S572	94%		
S573	94%		
S574	88%		
T607	59%		
T616	6%		
S618	18%		
S622	88%	CL5	
S627	18%		
S630	88%		
T632	47%		
S635	76%		
S653	59%		YES
S655	100%	CL6	
S656	100%	CL6	
S661	100%	CL7	
S667	100%	CL7	
