# Supplementary material for: Regulation of PDF receptor signaling controlling daily locomotor rhythms in Drosophila
Source: PLoS Genet. 2022 May 23;18(5):e1010013. doi: 10.1371/journal.pgen.1010013 (PMC9166358; doi:10.1371/journal.pgen.1010013)
Supplement: S4 Table — (PDF) [file pgen.1010013.s004.pdf]

S4 Table. Phosphopeptides derived from PDFR-Tandem, detected *in vivo*

Spectra are shown in S14_Figure

Experiment	collection time	phosphorylated residue	Tryptic Peptide	CL #
2	evening	S531	(R)AS*MYSGAYNTAPDTAVQPAGDPSATGK	CL2
3	evening	S531	(R)AS*MYSGAYNTAPDTAVQPAGDPSATGK	CL2
		T543	(R)ASMYSGAYNTAPDT*AVQPAGDPSATGK	CL3
		S560	(K)RIS*PPNKR	---
5	evening	S531	(R)AS*MYSGAYNTAPDTAVQPAGDPSATGK	CL2
		S534	(R)ASMYS*GAYNTAPDTAVQPAGDPSATGK	CL2
7	morning	S653	(R)VPS*ASSVPPESVVFELSEQGPVAIEFGSWSHPQFEK	---
8	evening	S531	(R)AS*MYSGAYNTAPDTAVQPAGDPSATGK	CL2
		S560	(K)RIS*PPNKR	---
