# Supplementary material for: Regulation of PDF receptor signaling controlling daily locomotor rhythms in Drosophila
Source: PLoS Genet. 2022 May 23;18(5):e1010013. doi: 10.1371/journal.pgen.1010013 (PMC9166358; doi:10.1371/journal.pgen.1010013)
Supplement: S5 Table — (PDF) [file pgen.1010013.s005.pdf]

S5 Table. Rhythmic behavior under Constant Dark Conditions
for flies in which *GRK1*, *GRK2* and *KRZ* (β-arrestin2) are manipulated

Genotype	Fly #	AR%	TAU	PWR	WI	SNR	T-test
timg > +	16	0%	24.28	157.71	6.13	1.863	
timg > gprk1	15	20%	24.42	49.17	4.17	0.541	ns
timg > gprk2.2	15	47%	24.56	53.83	4.00	0.616	ns
dcr2; timg > +	16	0%	24.34	193.14	6.19	2.784	
dcr2; timg > gprk1 RNAi	15	53%	23.86	62.70	4.43	0.583	ns
dcr2; timg > gprk2 RNAi	15	13%	24.38	73.25	3.92	0.864	ns
dcr2; tim >+	11	0%	24.05	123.30	7.18	2.69	
dcr2; timg > KK10463 gprk2 RNA	15	7%	23.57	64.76	4.50	0.84	***
dcr2; timg > KK103756 krz RNAi	14	100%	---	---	---	---	---

tau values compared to controls with Student's t-Test:

ns - not different;

*** - different, p < 0.01
