# Supplementary material for: Regulation of PDF receptor signaling controlling daily locomotor rhythms in Drosophila
Source: PLoS Genet. 2022 May 23;18(5):e1010013. doi: 10.1371/journal.pgen.1010013 (PMC9166358; doi:10.1371/journal.pgen.1010013)
Supplement: S1 Fig — The predicted 7th transmembrane domain (TM7) is marked in GREY. The C terminal tail starts with V505 (numbering for the melanogaster protein). The 28 potentially phosphorylated residues in the D.m. C terminal tail are highlighted in color: the 14 residues chosen for analysis are marked by their Cluster (CL) designation (1 to 7) and marked in AQUA; the 14 non-selected residues are marked in YELLOW. See S2 Table for additional sequence information. (PDF) [file pgen.1010013.s006.pdf]

S1 Fig

- (grey) TM7 – Transmembrane Segment 7
- (cyan) Conserved Ser/Thr/Tyr; Modified in Experiments
- (yellow) Conserved Ser/Thr/Tyr; Not Modified in Experiments

```
                     -----TM7--------     CL1   CL1         CL2-  -CL3
melanogaster   FAVWSYGTHFLTSFQGFFIALIYCFLNGEVRAVLLKSLATQLSVRGHPEWAPKRASMYSGAYNTAPDTDAV--QPAGD---   552
takahashi      FAVWSYVTHFLTSFQGFFIALIYCFLNGEVRAVLLKSLATQLSVRGHPEWAPKRASMYSGAYNTAPDTDAV--QPAGD---   619
ficusphila     FAVWSYGTHFLTSFQGFFIALIYCFLNGEVRAVLLKSLATQLSVRGHPEWAPKRASMYSGAYNTAPDTDAV--HPAGD---   608
rhopaloa       FAVWSYGTHFLTSFQGFFIALIYCFLNGEVRAVLLKSLATQLSVRGHPEWAPKRASMYSGAYNTAPDTDAV--QPAGD---   599
eugracilis     FAVWSYGTHFLTSFQGFFIALIYCFLNGEVRAVLLKSLATQLSVRGHPEWAPKRASMYSGAYNTAPDTDAV--QPAGD---   605
erecta         FAVWSYGTHFLTSFQGFFIALIYCFLNGEVRAVLLKSLATQLSVRGHPEWAPKRASMYSGAYNTAPDTDAV--QPAGD---   602
simulans       FAVWSYGTHFLTSFQGFFIALIYCFLNGEVRAVLLKSLATQLSVRGHPEWAPKRASMYSGAYNTAPDTDAV--QPAGD---   643
persimilis     FAVWSYGTHFLTSFQGFFIALIYCFLNGEVRACLLKSLSNQLSLRGHPEWAPKRPSMYSGAYNTAPDTDAVGQQPVAETQ-   525
miranda        FAVWSYGTHFLTSFQGFFIALIYCFLNGEVRACLLKSLSNQLSLRGHPEWAPKRPSMYSGAYNTAPDTDAVGQQPVAETQ-   619
willistoni     FAVWSYGTHFLTSFQGFFIALIYCFLNGEVRAVLLKSLATQMSVRGHPEWVPKRASMYSGAYNTAPDTDAVQTQPQGGPDI   605
bipectinata    FAVWSYGTHFLTSFQGFFIALIYCFLNGEVRAVLLKSLATQMSVRGHPEWVPKRASMYSGAYNTAPDTDAVIQQP-GD---   626
albomicans     FAVWSYVTHFLTSFQGFFIALIYCFLNGEVRTVLLKSLAVWMSVRGHPEWVPKRASMYSGAYNTAPDTDAL-QQP-GDAPP   641
grimshawi      FAVWSYVTHFLTSFQGFFIALIYCFLNGEVRTVLLKSLAVWMSVRGHPEWAPKRASMYSGAYNTAPDTDVV-QQP-GE---   623
virilis        FAIWSYVTHFLTSFQGFFIALIYCFLNGEVRAVMLKSIAVWLSVRGHPEWAPKRPSMYSGAYNTAPDTDPQLKQ--GD---   473
hydei          FAIWSYVTHFLTSFQGFFIALIYCFLNGEVRAVLLKSLAVWMSVRGHPEWVPKRASMYSAAYNTAPDTEAPAQQT-VD---   601
novamexicana   FAIWSYVTHFLTSFQGFFIALIYCFLNGEVRAVMLKSIAVWLSVRGHPEWAPKRPSMYSGAYNTAPDTDPQLKP--GD---   581
navajoa        FAIWSYVTHFLTSFQGFFIALIYCFLNGEVRAVLLKSLAVWMSVRGHPEWIPKRASMYSAAYNTAPDTEPPAQQS-VE---   602

                      CL4
melanogaster   PSATG---KRISPPNKRLNGRKPSSASIVMIHEPQQRQRLMRLMPRLQNKAREKGKD--RVEK----TDAEA---------   614
takahashi      PSAT----KRISPPHKRLNGRKPSSASIVMIHEPQQRQRLIRLIPRLQNKAREKSRD--RVDKADAETDPQA---------   633
ficusphila     PSAPG---KRISPPNKRLNGRKPSSASIVMIHEPQQRHRLIRLIPRLQNKNQEKGKD--RVEKADIDTEL-----------   623
rhopaloa       PLATG---KRISPPNKRLNGRKPSSASIVMIHEPQQRQRLIRLIPRLQNKAREKGRD--RVEKADAETEPEP---------   612
eugracilis     PSATG---KRISPPNKRLNGRKPSSASIVMIHEPQQRQRLIRLIPRLQNKAREKSKD--RVEKAEPETEP-----------   620
erecta         PSATG---KRISPPNKRLNGRKPSSASIVMIHEPQQRQRLMRLMPRLQNKAREKGKE--RVEK----TDKEA---------   616
simulans       PSATG---KRISPPNKRLNGRKPSSASIVMIHEPQQRQRLMRLMPRMQNKAREKGKD--RVEK----TDAEA---------   660
persimilis     -AATGG--LRTSPINRR-QN---TSASIVMIHEPNHRQRLVRLVQRNHNNNNQESPRRQRGER----TDDEDRIGIAAGAG   532
miranda        -AATGG--LRTSPINRR-QN---TSASIVMIHEPNHRQRLVRLVQRNHNNNNQESPRRQRGER----TDDEDRIGIAAGAG   626
willistoni     LPSTG---KRISPPNKRHTSRKASSASIVIIHEPQQRQRLLRLLQRSSTMNISNAKNLTNQT-----TDIDPEAPGVSPTA   608
bipectinata    NPASG---KRISPPNKRLNGRKASSASIVMIHEPQQRHRLLRLLPRLHSQRKDRDKIKDRDKDNDRDRQRDDRQLE-----   629
albomicans     PATTTAAGKRVSPPNKRLNCRKASSVSIVIAKEPQRQRLVQLVQQQQQQQRNNNN-SRNIMPDDE----------ASAS-   653
grimshawi      AISTS---RRVPPPIKRLKSRKANNVTIVISNEPQQQQQHQMLQQRNTNNNSTNGSSPRTV--EDDTGS------AMAT-   634
virilis        PQQSG---KRLSQSTKRSNSRKASSVTIVISTEPQIHRYVPYVPRRNNNNRASTGSA--RVRGILKATEEP-----ASGSA   573
hydei          ALSSS---LRISPQNRRLNSRKASSVIIVIANEPQRQRFVQFVQQRNNNNTANSN----------QGKDEP-----ASGSA   619
novamexicana   PQQSG---KRLSQSTKRLNSRKASSVTIVISTEPQIHRYIPYIPRRNNNNRASTDSA--RVRGILKATEEP-----ASGSA   571
navajoa        ALSSN---IRISPQSRRLNCRKASSVIIVIANEPQRQRSVQSVQQRNNNNTANGNGN--------QGKDEP-----ASGSA   618

                      CL5                                               CL6   CL7    CL7
melanogaster   --EPDPTISHIHSKEAG------SARSR-TRGSKWIMG-ICFRGQKVLRVPSA-SSVPPESVVFELSEQ     669
takahashi      ----DPAISRIHSKESSGGGGG-STGSR-NRGSKWIMG-ICFRGQKVLRVPSA-SSVPPESVVFELSEQ     693
ficusphila     ----DPAITRIQSKEST------GSTSR-SRGSKWIMG-ICFRGQKVLRVPSA-SSVPPESVVFELSEQ     679
rhopaloa       ----DPAITRIHSKEAGSTG---GTASRNSRGSKWIMG-ICFRGQKVLRVPSA-SSVPPESVVFELSEQ     671
eugracilis     ----DPAISRIHSKETG------VGGSR-TRGSKWIMG-ICFRGQKVLRVPSA-SSVPPESVVFELSEQ     675
erecta         EPEPDPAISRIHSKEAD------RARSR-TRGSKWIMG-ICFRGQKVLRVPSA-SSVPPESVVFELSEQ     671
simulans       ----EPAIARIHSKEAG------SARSR-TRGSKWIMG-ICFRGQKVLRVPSA-SSVPPESVVFELSEQ     715
persimilis     -VEAEVVVV-QDSVGGAVG---RKRETRIHAGTKWMISGLCFRGQKVLRVPST-SSVPPESVVFELSEQ     591
miranda        -VEAEVVVV-QDSVGGAVG---RKRETRIHAGTKWMISGLCFRGQKVLRVPST-SSVPPESVVFELSEQ     685
willistoni     MMDANAATQRIHSKESS-------ARTNST--PNWMIFVLCFRGQKVLRVPPASSVPPESVVFELSRQ     663
bipectinata    ETEGDPATTRIHSKETAR--------TGRNRGSKWMMDIICFRGQKVLRVPSA-SSVPPESVVFELSEQ     684
albomicans     -------ATRIQIKDA-----------PSSGHRNWMIG-LCFRGQKVLRVPPA-SSVPPESVVFELSEQ     702
grimshawi      ---------RIRSKES------------SAGRSNWMTN-LCFRGKKVLRVPPA-SSVPPESVVFELSEL     679
virilis        ------VGQRIRSTDDG----------TG--RNSNWMFG-LCFRGQKVLRVPPA-SSVPPESVVFELSEQ     623
hydei          ---------RIRGKEA--------AST-ARNNGNWKFS-LCFRGQKVLRVPPA-SSVPPESVVFELSEQ     668
novamexicana   ------VGQRIRSTDDG-------PDTG--RNSNWMFG-LCFRGQKVLRVPPA-SSVPPESVVFELSEQ     623
navajoa        -----RSGQRIRSKE-T--------ASTG-RSTGNWMFS-LCFHGQKVLRVPPA-SSVPPESVVFELSEQ     671
```

S1 Fig. Alignment of the C terminal PDFR-A sequences from 17 different *Drosophalid* species. The predicted 7th transmembrane domain (TM7) is marked in GREY. The C terminal tail starts with V505 (numbering for the *melanogaster* protein). The 28 potentially phosphorylated residues in the D.m. C terminal tail are highlighted in color: the 14 residues chosen for analysis are marked by their Cluster (CL) designation (1 to 7) and marked in AQUA; the 14 non-selected residues are marked in YELLOW. See **S2 Table** for additional sequence information.
