# Supplementary material for: Regulation of PDF receptor signaling controlling daily locomotor rhythms in Drosophila
Source: PLoS Genet. 2022 May 23;18(5):e1010013. doi: 10.1371/journal.pgen.1010013 (PMC9166358; doi:10.1371/journal.pgen.1010013)
Supplement: S6 Fig — All behavioral records were recorded from han (pdfr mutant) flies that expressed either no UAS transgene (A), or a UAS-WT pdfr transgene (B) or a variety of Simple pdfr Variants, including 2-3A (C), 4A (D), 5A (E), 6A (F) or 7A (G), or Multiple pdfr variants, including 5-7A (H), 1-4A (I), 1-5A (J), and 1-6A (K). Top Right Box: A schematic of the PDFR C terminal segment for the WT and all variants studied: see Figure Legend 1 for details. Letters to the right of each variant C terminal segment correspond to the Panels in this Figure that display the behavior observed following its expression. Each Panel (A)-(L) contains sub-panels (1) and (2): Sub-Panel (1) displays a daily plot of locomotor activity (a group eduction) averaged over the last two days of entrainment (LD 5–6). Open bars indicate the 16 hr periods of Lights-on and filled bars indicate 8 hr periods of Lights-off. Sub-panel (2) displays a double-plotted group actogram throughout the 6 days of LD entrainment, followed by ~9 days of (DD, grey background). Green lines indicate the phase of the dominant activity period in DD. Panel L displays the average Morning activity Phase Offset timepoint, and Panel M displays the average Evening activity Phase Onset (marked by a Blue Arrow) for each genotype over the last two days of entrainment (LD 5–6). The positions of the Red and Blue arrow in panels A-K are representative phase points; panels L and M present their true values respectively. Analyses represent ANOVA followed by Dunnett’s post hoc multiple comparisons of all compared to WT: ns = not significant; * = p<0.05; ** = p<0.01; *** = p<0.005; **** = p<0.001. (PDF) [file pgen.1010013.s011.pdf]

Supplemental Figure 6

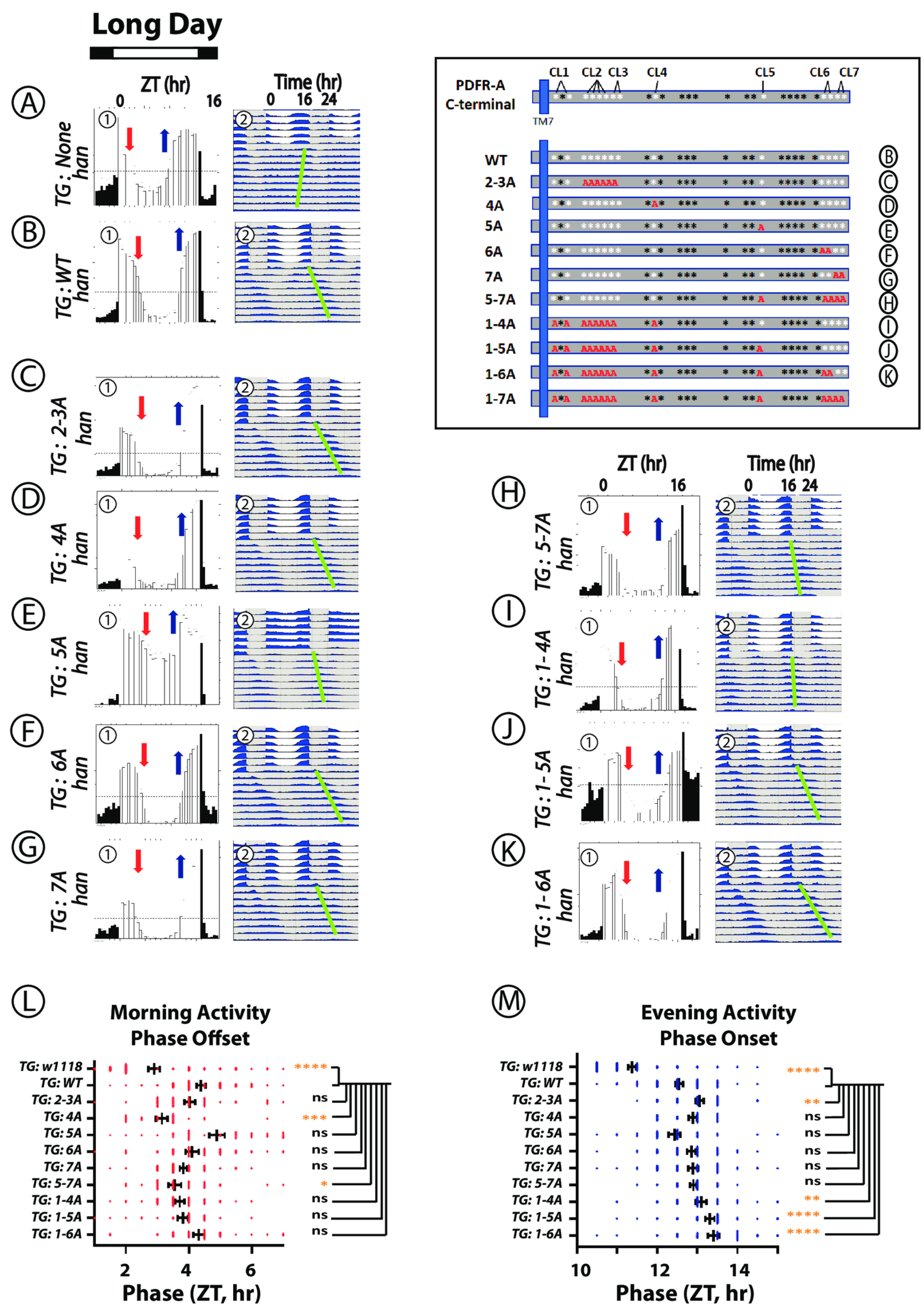

S6 Fig. Locomotor Rhythms exhibited by WT PDFR and by other PDFR Variants under Long-day (summer-like) condition. All behavioral records were recorded from *han* (*pdfr* mutant) flies that expressed either no UAS transgene (A), or a UAS-WT *pdfr* transgene (B) or a variety of Simple *pdfr* Variants, including 2-3A (C), 4A (D), 5A (E), 6A (F) or 7A (G), or Multiple *pdfr* variants, including 5-7A (H), 1-4A (I), 1-5A (J), and 1-6A (K). <u>Top Right Box:</u> A schematic of the PDFR C terminal segment for the WT and all variants studied: see Figure Legend 1 for details. Letters to the right of each variant C terminal segment correspond to the Panels in this Figure that display the behavior observed following its expression. Each Panel (A)-(L) contains sub-panels (1) and (2): Sub-Panel (1) displays a daily plot of locomotor activity (a group eduction) averaged over the last two days of entrainment (LD 5-6). Open bars indicate the 16 hr periods of Lights-on and filled bars indicate 8 hr periods of Lights-off. Sub-panel (2) displays a double-plotted group actogram throughout the 6 days of LD entrainment, followed by ~9 days of (DD, grey background). Green lines indicate the phase of the dominant activity period in DD. Panel L displays the average Morning activity Phase Offset timepoint, and Panel M displays the average Evening activity Phase Onset (marked by a Blue Arrow) for each genotype over the last two days of entrainment (LD 5-6). The positions of the Red and Blue arrow in panels A-K are representative phase points; panels L and M present their true values respectively. Analyses represent ANOVA followed by Dunnett's post hoc multiple comparisons of all compared to WT: ns = not significant; * = $p<0.05$; ** = $p<0.01$; *** = $p<0.005$; **** = $p<0.001$.
