# Supplementary material for: Regulation of PDF receptor signaling controlling daily locomotor rhythms in Drosophila
Source: PLoS Genet. 2022 May 23;18(5):e1010013. doi: 10.1371/journal.pgen.1010013 (PMC9166358; doi:10.1371/journal.pgen.1010013)
Supplement: S7 Fig — Behavioral records recorded from han (pdfr mutant) flies that expressed either no UAS transgene (A–marked in YELLOW), or a WT pdfr cdNA (B–marked in BLACK) or a variety of Simple pdfr Variants (all marked in RED) including 2-3A (C), 4A (D), 5A (E), 6A (F), 7A (G), 5-7A (H), 1-4A (I), 1-5A (J), and 1-6A (K). These measures were averaged across all experiments run for each individual genotype (see Table 1 for N and n values). Top Right Box: A schematic of the PDFR C terminal segment for the WT and all variants studied: see Figure Legend 1 for details. Letters to the right of each variant C terminal segment correspond to the Panels in this Figure that display the behavior observed following its expression. Each Panel (A)-(L) contains sub-panels (1) through (3), each of which displays Bin-by-Bin analyses of activity levels sorted by 30 min bins, for three different time periods: (1) ZT17-23.5; (2) ZT 0.5–8; (3) ZT 8.5–16. The missing bin at timepoint 0 contains the startle response that accompanies the sudden lights-on signal. Blue asterisks indicate significantly-different activity levels according to a Student’s T-test following an ANOVA (p< 0.05). Red arrows highlight elevated Morning activity levels displayed by different PDFR variants. Blue arrows highlight elevated Evening activity levels produced by different PDFR variants. (PDF) [file pgen.1010013.s012.pdf]

Supplemental Figure 7.

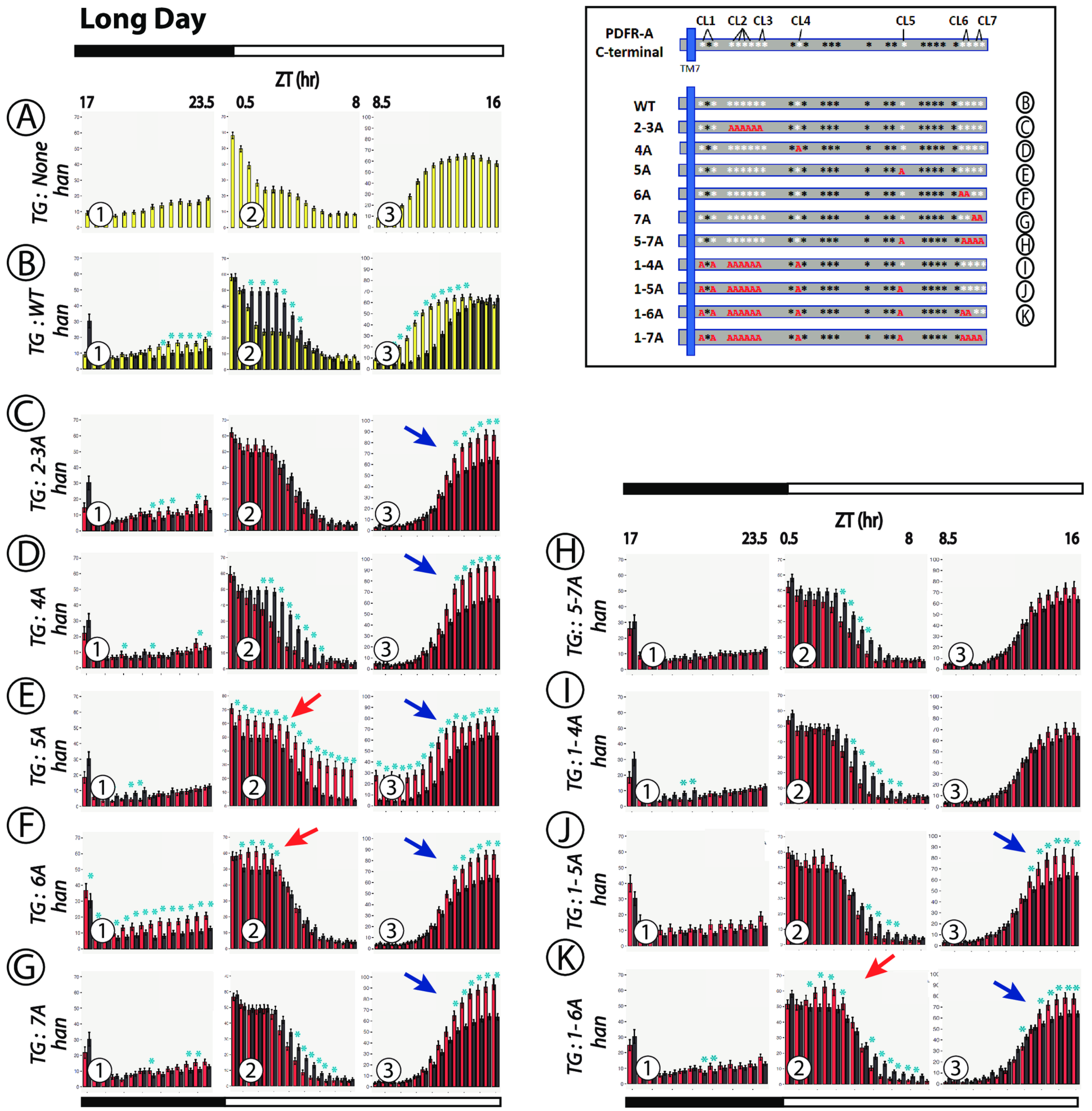

S7 Fig. Amplitude measures of locomotor rhythms exhibited by WT PDFR and by other PDFR Variants under Long-day (summer-like) conditions. Behavioral records recorded from *han* (*pdfr* mutant) flies that expressed either no UAS transgene (A – marked in YELLOW), or a WT *pdfr* cdNA (B – marked in BLACK) or a variety of Simple *pdfr* Variants (all marked in RED) including 2-3A (C), 4A (D), 5A (E), 6A (F), 7A (G), 5-7A (H), 1-4A (I), 1-5A (J), and 1-6A (K). These measures were averaged across all experiments run for each individual genotype (see Table 1 for N and n values). <u>Top Right Box:</u> A schematic of the PDFR C terminal segment for the WT and all variants studied: see Figure Legend 1 for details. Letters to the right of each variant C terminal segment correspond to the Panels in this Figure that display the behavior observed following its expression. Each Panel (A)-(L) contains sub-panels (1) through (3), each of which displays Bin-by-Bin analyses of activity levels sorted by 30 min bins, for three different time periods: (1) ZT17-23.5; (2) ZT 0.5 – 8; (3) ZT 8.5-16. The missing bin at timepoint 0 contains the startle response that accompanies the sudden lights-on signal. Blue asterisks indicate significantly-different activity levels according to a Student's T-test following an ANOVA ($p < 0.05$). Red arrows highlight elevated Morning activity levels displayed by different PDFR variants. Blue arrows highlight elevated Evening activity levels produced by different PDFR variants.
