# Supplementary material for: Regulation of PDF receptor signaling controlling daily locomotor rhythms in Drosophila
Source: PLoS Genet. 2022 May 23;18(5):e1010013. doi: 10.1371/journal.pgen.1010013 (PMC9166358; doi:10.1371/journal.pgen.1010013)
Supplement: S8 Fig — All behavioral records were recorded from han (pdfr mutant) flies that expressed either no UAS transgene (panels (A, D, I, and L)—yellow), or a WT pdfr cdNA (panels (B, E, J and M)—black) or the PDFR 1-7A Multiple Variant (panels (C, F, K and N)–red). Panels (A-F) present data recorded under Short-Day (winter-like) conditions; panels (I-N) present data recorded under Long Day (summer-like) conditions. Each Panel (A—C) and (I -K) contains four sub-panels: Sub-Panel (1) displays an average daily plot of locomotor activity averaged over the final two days of light entrainment (a group eduction): Open bars indicate the periods of Lights-on and filled bars indicate periods of Lights-off. Sub-Panels 2–4 display Bin-by-Bin analyses of activity levels sorted by 30 min bins, for three different time periods: (1) ZT17-23.5; (2) ZT 0.5–8; (3) ZT 8.5–16. The missing bin at timepoint 0 contains the startle response that accompanies the sudden lights-on signal. Blue asterisks indicate significantly-different activity levels according to a Student’s T-test following an ANOVA (p< 0.05). Panels (D—F) and (L—N) display double-plotted group actograms throughout the 6 days of Light: dark entrainment, followed by 9 days of constant darkness (DD, grey background). Panels (G) and (H) display the average Phase Onsets and Offsets (respectively) for the Morning and Evening activity periods for each genotype over the last two days of entrainment under short days (LD 5–6). Blue arrows highlight the elevated Evening activity amplitudes. Panels (O) and (P) display the average Phase Offsets and Onsets (respectively) for the Morning and Evening activity periods for each genotype over the last two days of entrainment under long days (LD 5–6). Red Arrows highlight the elevated amplitude of the Morning peak. Ns–not significant; *—p < 0.05; ***—p < 0.005; ****—p < 0.001. (PDF) [file pgen.1010013.s013.pdf]

S8 Fig

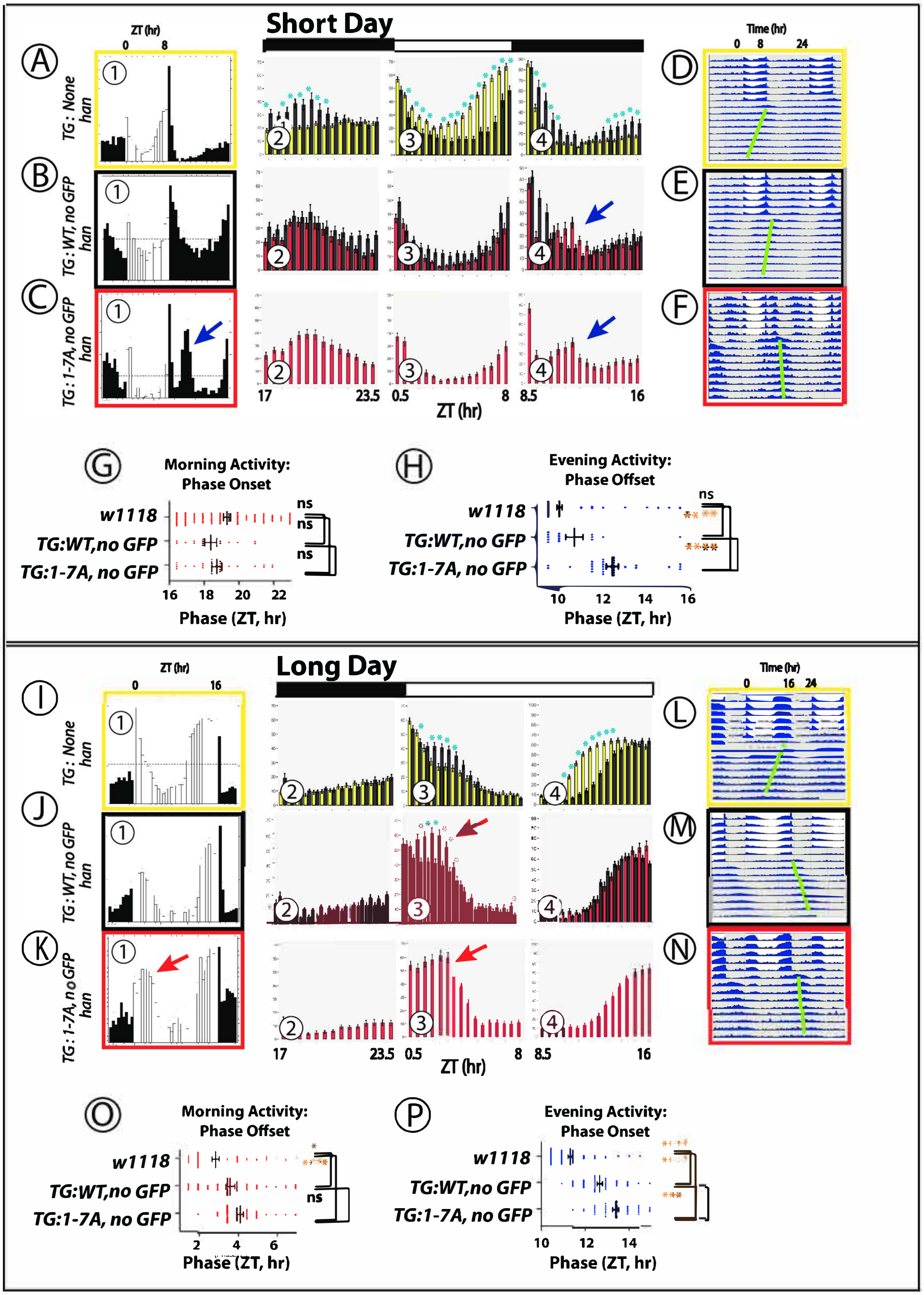
Short Day
Long Day
ZT (hr)
Time (hr)
TG: None han
TG: WT, no GFP han
TG: 1-7A, no GFP han
Morning Activity: Phase Onset
Evening Activity: Phase Offset
Morning Activity: Phase Offset
Evening Activity: Phase Onset
w1118
TG:WT,no GFP
TG:1-7A, no GFP
ns
Phase (ZT, hr)

S8 Fig. Average daily locomotor rhythms in flies expressing WT and 1-7A variant PDFR transgenes that lack GFP fusions. All behavioral records were recorded from *han* (*pdfr* mutant) flies that expressed either no UAS transgene (panels (A, D, I, and L) - yellow), or a WT *pdfr* cdNA (panels (B, E, J and M) - black) or the PDFR 1-7A Multiple Variant (panels (C, F, K and N) – red). Panels (A-F) present data recorded under Short-Day (winter-like) conditions; panels (I-N) present data recorded under Long Day (summer-like) conditions. Each Panel (A - C) and (I -K) contains four sub-panels: Sub-Panel (1) displays an average daily plot of locomotor activity averaged over the final two days of light entrainment (a group eduction): Open bars indicate the periods of Lights-on and filled bars indicate periods of Lights-off. Sub-Panels 2-4 display Bin-by-Bin analyses of activity levels sorted by 30 min bins, for three different time periods: (1) ZT17-23.5; (2) ZT 0.5 – 8; (3) ZT 8.5-16. The missing bin at timepoint 0 contains the startle response that accompanies the sudden lights-on signal. Blue asterisks indicate significantly-different activity levels according to a Student's T-test following an ANOVA ($p < 0.05$). Panels (D - F) and (L - N) display double-plotted group actograms throughout the 6 days of Light: dark entrainment, followed by 9 days of constant darkness (DD, grey background). Panels (G) and (H) display the average Phase Onsets and Offsets (respectively) for the Morning and Evening activity periods for each genotype over the last two days of entrainment under short days (LD 5-6). Blue arrows highlight the elevated Evening activity amplitudes. Panels (O) and (P) display the average Phase Offsets and Onsets (respectively) for the Morning and Evening activity periods for each genotype over the last two days of entrainment under long days (LD 5-6). Red Arrows highlight the elevated amplitude of the Morning peak. Ns – not significant; * - $p < 0.05$; *** - $p < 0.005$; **** - $p < 0.001$
