# Supplementary material for: Regulation of PDF receptor signaling controlling daily locomotor rhythms in Drosophila
Source: PLoS Genet. 2022 May 23;18(5):e1010013. doi: 10.1371/journal.pgen.1010013 (PMC9166358; doi:10.1371/journal.pgen.1010013)
Supplement: S9 Fig — Luciferase measurements in hEK-293T cells stably expressing WT PDFR or its variants and transiently expressing CRE-Luciferase. The histogram represents basal levels of 2nd messenger signaling, i.e., in the absence of stimulation by neuropeptide PDF. Values represent the mean +/-SEM of three independent measurements, and were analyzed by Student ‘s T-test: * = p < 0.05; ns = not significantly different. (PDF) [file pgen.1010013.s014.pdf]

S9 Fig

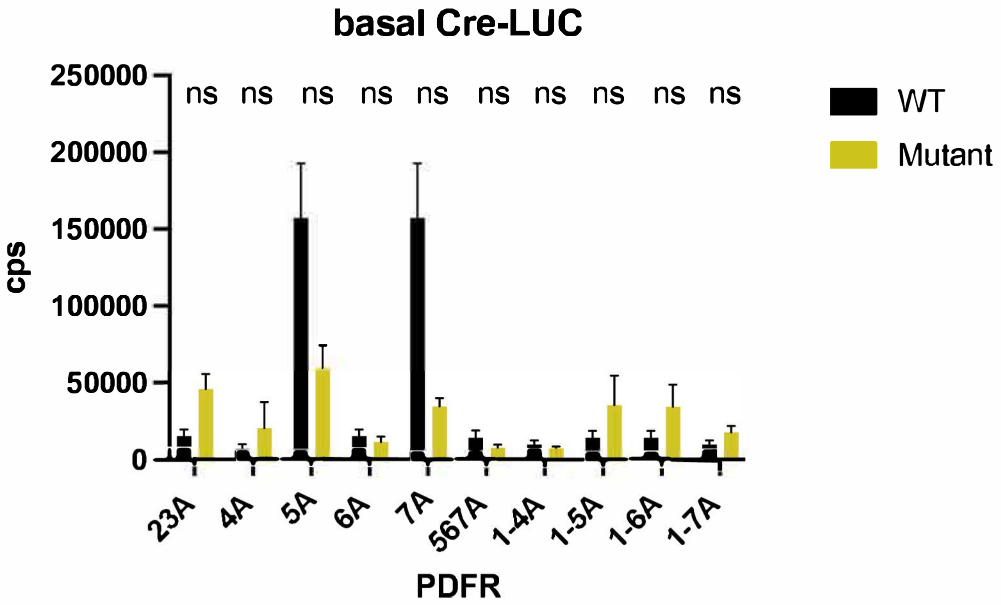

basal Cre-LUC
250000
200000
150000
100000
50000
0
cps
ns
23A
4A
5A
6A
7A
567A
1-4A
1-5A
1-6A
1-7A
PDFR
WT
Mutant

S9 Fig. Basal cAMP signaling displayed by the PDFR variant series following functional expression *in vitro*. Luciferase measurements in *hEK-293T* cells stably expressing WT PDFR or its variants and transiently expressing *CRE-Luciferase*. The histogram represents basal levels of 2nd messenger signaling, i.e., in the absence of stimulation by neuropeptide PDF. Values represent the mean +/-SEM of three independent measurements, and were analyzed by Student 's T-test: * = $p < 0.05$; ns = not significantly different.
