# Supplementary material for: Regulation of PDF receptor signaling controlling daily locomotor rhythms in Drosophila
Source: PLoS Genet. 2022 May 23;18(5):e1010013. doi: 10.1371/journal.pgen.1010013 (PMC9166358; doi:10.1371/journal.pgen.1010013)
Supplement: S12 Fig — β -Lactamase activity measurements in hEK-293T cells stably expressing WT PDFR or its variants fused to β -lactamase at the N terminus. The histograms represent the basal values for surface receptor expression in the absence of stimulation by neuropeptide PDF. Values represent the mean +/-SEM of three independent measurements, and were analyzed by Student ‘s T-test: * = p < 0.05; ns = not significantly different. (PDF) [file pgen.1010013.s017.pdf]

S12 Fig

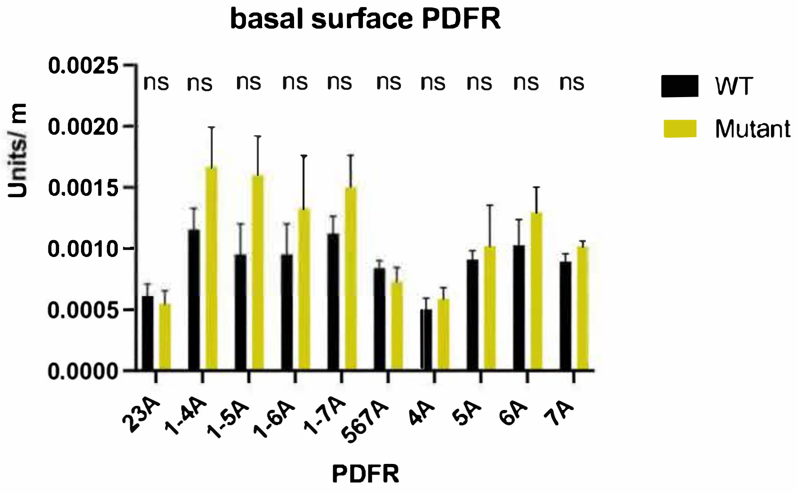
basal surface PDFR
Units/ m
0.0025
0.0020
0.0015
0.0010
0.0005
0.0000
ns ns ns ns ns ns ns ns ns ns
WT
Mutant
23A
1-4A
1-5A
1-6A
1-7A
567A
4A
5A
6A
7A
PDFR

S12 Fig. **Surface expression of the PDFR variant series following functional expression *in vitro***. β -Lactamase activity measurements in *hEK-293T* cells stably expressing WT PDFR or its variants fused to β -lactamase at the N terminus. The histograms represent the basal values for surface receptor expression in the absence of stimulation by neuropeptide PDF. Values represent the mean +/-SEM of three independent measurements, and were analyzed by Student ‘s T-test: * = $p < 0.05$; ns = not significantly different.
