# Supplementary material for: Regulation of PDF receptor signaling controlling daily locomotor rhythms in Drosophila
Source: PLoS Genet. 2022 May 23;18(5):e1010013. doi: 10.1371/journal.pgen.1010013 (PMC9166358; doi:10.1371/journal.pgen.1010013)
Supplement: S13 Fig — β-Lactamase activity measurements in hEK-293T cells stably expressing WT PDFR or its variants fused to β -lactamase at the N terminus. The histograms represent the values for surface receptor expression 20 m after exposure to neuropeptide PDF. Values represent the mean +/-SEM of three independent measurements, and were analyzed by Student ‘s T-test: * = p < 0.05; ns = not significantly different. (PDF) [file pgen.1010013.s018.pdf]

S13 Fig

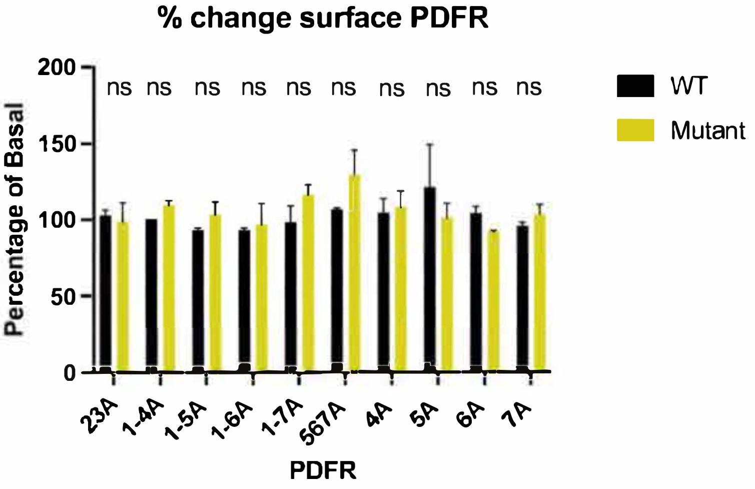
% change surface PDFR
Percentage of Basal
200
150
100
50
0
ns ns ns ns ns ns ns ns ns ns
WT
Mutant
23A
1-4A
1-5A
1-6A
1-7A
567A
4A
5A
6A
7A
PDFR

S13 Fig. Percentage change in surface expression of the PDFR variant series after exposure to PDF, following functional expression *in vitro*. β-Lactamase activity measurements in *hEK-293T* cells stably expressing WT PDFR or its variants fused to β -lactamase at the N terminus. The histograms represent the values for surface receptor expression 20 m after exposure to neuropeptide PDF. Values represent the mean +/-SEM of three independent measurements, and were analyzed by Student 's T-test: * = $p < 0.05$; ns = not significantly different.
