# Supplementary material for: Regulation of PDF receptor signaling controlling daily locomotor rhythms in Drosophila
Source: PLoS Genet. 2022 May 23;18(5):e1010013. doi: 10.1371/journal.pgen.1010013 (PMC9166358; doi:10.1371/journal.pgen.1010013)
Supplement: S14 Fig — Nine spectra of phosphopeptides derived from a UAS-PDFR-Tandem construct from eight independent immunoprecipitation experiments. (PDF) [file pgen.1010013.s019.pdf]

S14 Fig

A. Exp 2
PDFR S531 CL2

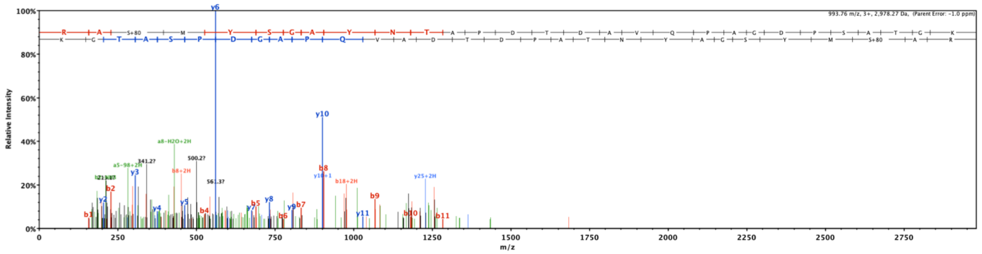

C. Exp. 3
PDFR T543 CL3

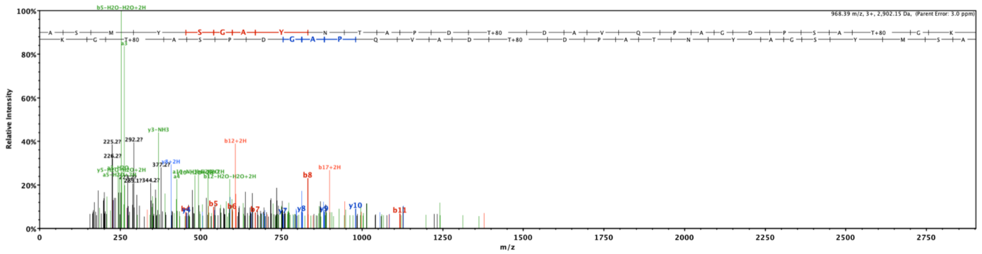

B. Exp. 3
PDFR S531 CL2

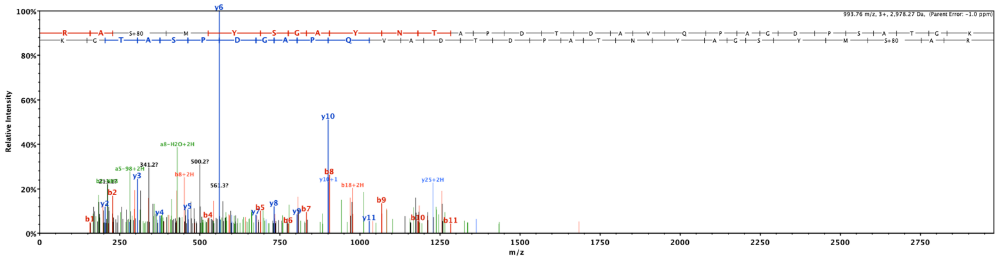

D. Exp 3
PDFR S560

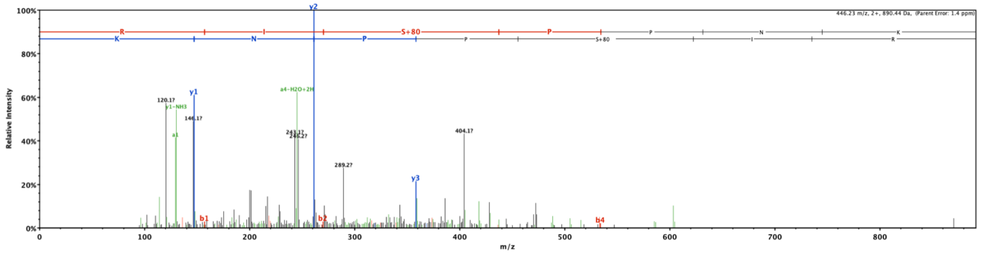

E. Exp. 5
PDFR – S531 CL2

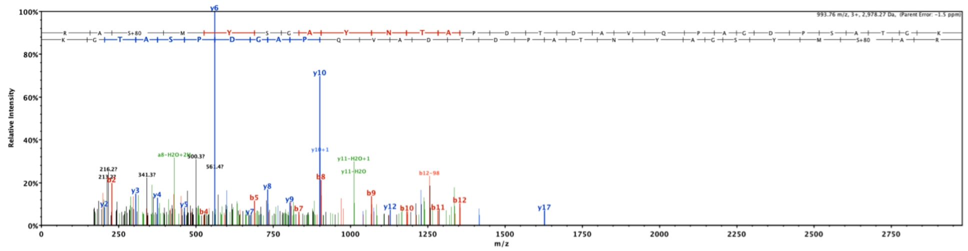

H. Exp. 7
PDFR S653

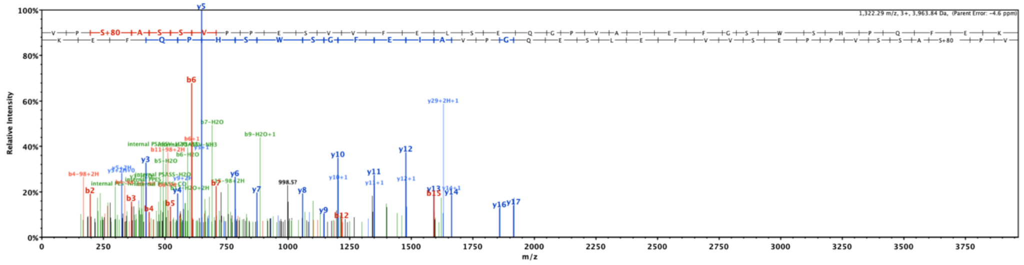

F. Exp. 5
PDFR - S534 CL2

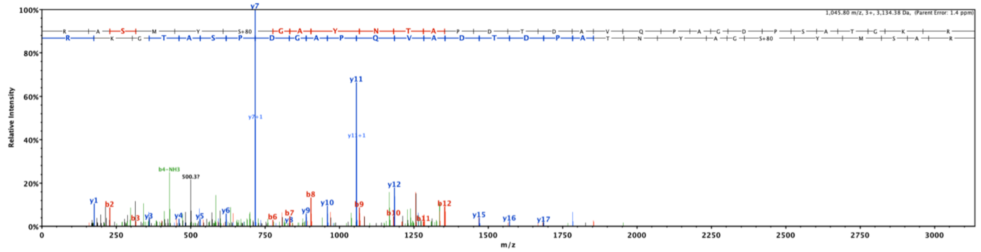

G. Exp 8
PDFR – S531 CL2

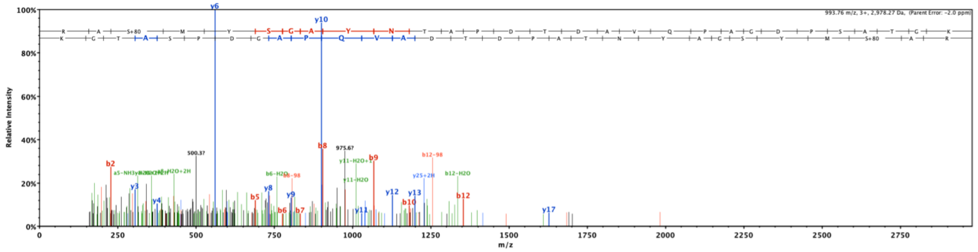

I. Exp. 8
PDFR – S560

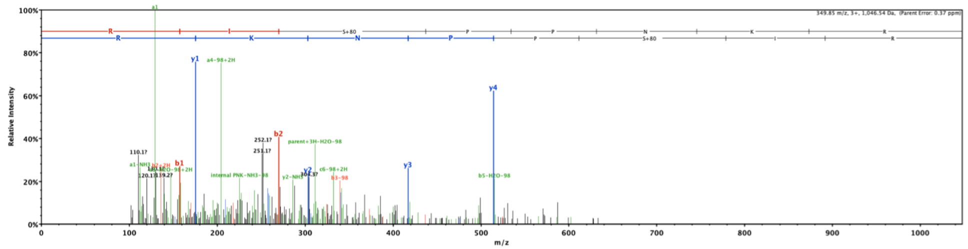

S14 Fig. *In vivo* detection of phosphopeptides from the PDFR C terminal tail. Nine spectra of phosphopeptides derived from a UAS-*PDFR*-Tandem construct from eight independent immunoprecipitation experiments.
