# Supplementary material for: Regulation of PDF receptor signaling controlling daily locomotor rhythms in Drosophila
Source: PLoS Genet. 2022 May 23;18(5):e1010013. doi: 10.1371/journal.pgen.1010013 (PMC9166358; doi:10.1371/journal.pgen.1010013)
Supplement: S15 Fig — Group eductions of locomotor activity profiles for different genotypes averaged over six days of light entrainment. Open bars indicate periods of Lights-On and filled bars indicate periods of Lights-Off. The column present manipulations for each of four different gene targets (none; Gprk1; Gprk2 and β-arr2 (kurtz)) using tim(UAS)-Gal4. The Upper panels (D and F) present over-expression experiments. The Middle (E and G) and Lower panels (H and I) present RNAi experiments. UAS-RNAi constructs illustrated in panels E and G were created and shared by the Paul Hardin laboratory. Values for N and n are found in S5 Table. (PDF) [file pgen.1010013.s020.pdf]

S15 Fig

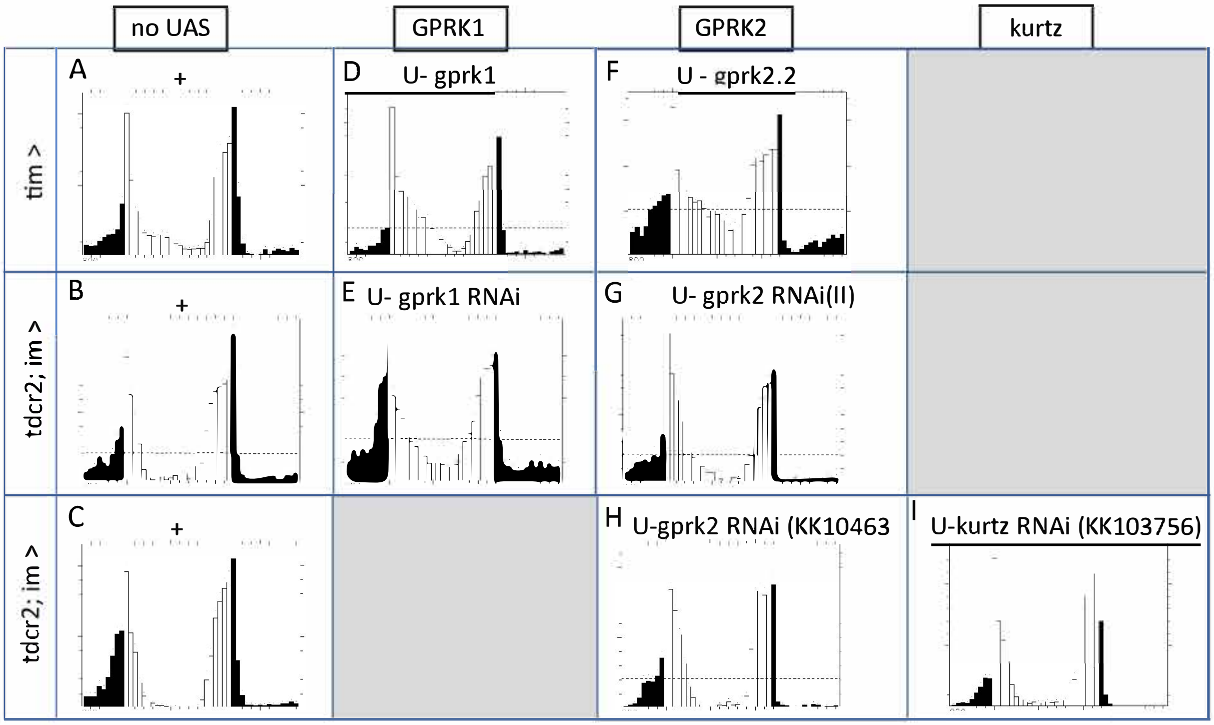
no UAS
GPRK1
GPRK2
kurtz
tim >
tdcr2; im >
tdcr2; im >
A
+
B
+
C
+
D
U- gprk1
E
U- gprk1 RNAi
F
U - gprk2.2
G
U- gprk2 RNAi(II)
H
U-gprk2 RNAi (KK10463
I
U-kurtz RNAi (KK103756)

S15 Fig. Average daily locomotor rhythms in flies with manipulations of *GRK1, GRK2* and *⍰-arrestin2*. Group eductions of locomotor activity profiles for different genotypes averaged over six days of light entrainment. Open bars indicate periods of Lights-On and filled bars indicate periods of Lights-Off. The column present manipulations for each of four different gene targets (none; *Gprk1*; *Gprk2* and ⍰-*arr2* ***(**kurtz))* using *tim*(UAS)-Gal4. The Upper panels (D and F) present over-expression experiments. The Middle (E and G) and Lower panels (H and I) present RNAi experiments. UAS-RNAi constructs illustrated in panels E and G were created and shared by the Paul Hardin laboratory. Values for N and n are found in S5 Table.
