# Supplementary material for: Regulation of PDF receptor signaling controlling daily locomotor rhythms in Drosophila
Source: PLoS Genet. 2022 May 23;18(5):e1010013. doi: 10.1371/journal.pgen.1010013 (PMC9166358; doi:10.1371/journal.pgen.1010013)
Supplement: S1 Text — (DOCX) [file pgen.1010013.s021.docx]

***SUPPORTING INFORMATION***

**Regulation of PDF receptor signaling controlling daily locomotor rhythms in *Drosophila*.**

***PDFR Isoforms***

Flybase reports evidence for 4 distinct *pdfr* RNAs and 3 PDFR protein isoforms – (http://flybase.org/cgi-bin/gbrowse2/dmel/?Search=1;name=FBgn0260753): together these encode either of two N terminal sequence variants (RA vs RB) and either of two different C terminal sequence variants (RA and RB vs. RC and RD). The C terminal sequence encoded by isoforms RC and RD replaces the final 20 residues encoded by RA and RB with an alternative 50 residues, by splicing to a downstream exon (#10). Exon #10 is specifically deleted in a fly stock we previously described (P2-1, containing a unilateral deletion of the entire Exon 10, derived from *P{BG12523}*: [1]. That deletion did not affect locomotor rhythmicity adversely, nor did it produce any other locomotor phenotypes that resembled those of the *han* loss of function *pdfr* alleles, which include deletions of the C terminus-encoding sequences of all isoforms. Hence, we experimentally considered the two RNA isoforms RA and RB which display the shorter C terminal tail. The RA isoform is represented in the construct UAS-*pdfr*-16 which we have used in other published studies (e.g., [1-4]. Therefore, we focused our experimental design on the contributions of the sequence features of the C terminal tail of the PDFR-PA protein isoform to the strength and time course of PDF signaling that shapes locomotor rhythms.

***Genomic sequence analysis***

We used the PDFR sequence *D. melanogaster* NP_570007.2 as a blastp query (<https://blast.ncbi.nlm.nih.gov/Blast.cgi?PAGE=Proteins>) to search among non-redundant protein sequences (nr). Species representatives were not accepted if the C terminal sequence was incomplete or if only non-PDFR-A isoforms were retrieved. Accession numbers for the 16 additional *Drosophalid* species that we accepted are listed in S1 Table. Sequences were aligned using Clustal (<https://www.ebi.ac.uk/Tools/msa/clustalo/>) followed by minor manual adjustments.

***DNA Construction***

**Primers.** All primers used in creating DNA constructs are listed in S2 Table.

**β-lactamase Fusions.** The expression vector β-lactamase: β-AR2 pcDNA3.1 was provided by Dr. Ali Salahpour [5, 6] and used to make a β-lactamase pcDNA3.1 vector. The *CG13758* cDNA [1] was cloned into the β-lactamase pcDNA3.1 vector in frame with β-*lac* sequence, with restriction sites AscI and Not1. We also cloned the β *lac* sequence into a pcDNA5/frt vector via Nhe1 and HindIII. The *pdfr* wild type and C-terminal mutation series (see C-terminal construct oligos) was cloned into β-lac pcDNA5/frt vector with HindIII and Not1.

**FLAG::PDFR::EGFP fusions.** The expression vector Flag CB1R EGFP-N1 was provided by Dr. Zsolt Lenkei [7]. 4xFlag CG13758 EGFP-N1 vector was constructed by cloning 3xFlag from p3xFLAG-CMV-14 vector (Millipore-Sigma) in frame with the 1xFlag, with restriction site BglII on N-terminus and BamHI-BglII on C-terminus. *CG13758* WT sequence was cloned in frame with 4xFlag at N-terminus and EGFP on the C-terminus, using restriction sites BglII and Age1 with the CG13758 PCR fragment and BamHI and Age1 with the 4xFlag EGFP vector. Finally, site-directed mutagenesis was performed to mutate potential C-terminal phosphorylation sites (see C-terminal construct oligos). All constructs from 4xFlag 13758 EGFP were transferred to pcDNA5/frt vector (using HindIII and Not1) to make stable cell lines and to UAS-*attb* vector (using EcoRI and Not1) to create transgenic *Drosophila* lines.

**PDFR-Tandem Fusions.** The 2007bp *pdfr* coding sequence was amplified from a pcDNA3.1 *pdfr* construct [1] and the stop codon removed. The primers used were, *pdfr*-F 5’-GGAGATCTGCCACCATGACCCTCCTGTCGAACATTCTCG-3’ and *pdfr*-R 5’-GGCGACCGGTCCCTGCTCTGACAACTCAAATACAACTGACTC-3’. The product was blunt end-cloned into Eco-RV-pBluescript. The 451bp Tandem tag fragment was amplified from pENTR tandem plasmid (kind gift of Dmitri Nusinow, Donald Danforth Plant Science Center) to include a stop site at its N-terminus using primers (Integrated DNA Technologies, Coralville, Iowa) Tandem-foward 5’-GGGGAATTCGGAAGCTGGAGCCACCCTCAATTTGAAAAGGG-3’ and Tandem-reverse 5’-CCTCTAGATTACTATCACTTCTCGAACTGAGGATGACTCCAAGATCC-3’, then ligated in-frame to the *Pdfr* gene by digesting both with EcoRI and XbaI. Following sequenced verification, the PDFR-Tandem was transferred to the BlII/XbaI-digested UAS-attb vector.

***β-Lactamase (βlac)-fusion protein*** ***Assay.***

To quantify PDFR surface expression in cell lines, we employed the β-Lactamase method [5, 6]. 4×10^4^ cells/well of *hEK-293* stably-expressing a β**-**lactamase-fusions to the C-termini of WT and sequence-variant PDFRs were split into a poly-lysine coated 96-well plate. Twenty-four hours after plating, the cells were incubated for 20 minutes at 37^o^C, with and without PDF peptide (Phoenix Pharmaceuticals, Inc., Burlingame, California) diluted in MEM to 10 ^-5^M. The cells were washed with PBS and treated with nitroceﬁn (Cayman Chemical, Ann Arbor, Michigan) diluted to a ﬁnal concentration of 100 μM in PBS. Immediately after adding the nitrocefin solution, the absorbance of each well was read at 490 nm every minute, for 30 min at 37^o^C, with a Synergy H4 Hybrid Multi-Mode microplate reader (Bio Tek Instruments, Inc., Winooski, VT). Change in absorbance per minute readings were calculated into linear slopes and R^2^ values. Basal levels of PDFR cell surface expression were obtained from cells not exposed to PDF. We divided the slope obtained from exposed cells exposed to peptide by the slope from cells not exposed to calculate % change in PDFR cell surface expression following 20 m exposure to PDF. Graphs and one-way ANOVA statistics were calculated using GraphPad Prism 8 software (San Diego, California).

***Tandem Affinity Purification.***

We grew *yw*; UAS-*pdfr*-tandem; *tim*-gal4 flies at 25°C on standard cornmeal medium.  Flies were collected on ice in 25 ml aliquots and stored at -80°C. Fly heads were collected by sieving, cooled in liquid nitrogen, ground in a Retsch 400 mixer mill (4 x; 30 Hz; 45 sec), and re-suspended in 5 ml 0.3% IGEPAL lysis buffer (50mM Tris-HCl pH7.5, 125mM NaCl, 5% Gycerol, IGEPAL 0.3%**,** 1.5mM MgCl_2_, 25mM NaF, 1mM Na_3_VO_4_, 0.05mM MG-115, 1mM PMSF, Protease inhibitor mix (Sigma P8340), Protease Inhibitor Cocktail (04693159001 Roche, Switzerland)) [8]. The head extract was sonicated (3-4 x; 10 sec), clarified by centrifugation at 4°C for 20 min at 50,000 x g and again for 40 minutes at 250,000 x g. The extract was then incubated with anti-flag M2 magnetic beads (M8823 Sigma, St. Louis, MO) on a rotator (1 hr at 4°C). The beads were washed twice in 0.3% IGEPAL lysis buffer, twice in Flag-to-HIS buffer (100 mM sodium phosphate, pH 8.0, 150 mM NaCl, 0.1% Triton X-100) and finally three times with Flag-to-HIS buffer without Triton X-100. The immunoprecipitated proteins were eluted from the anti-flag beads twice, at 4°C and then also at 30°C, using 500μg/ml 3x FLAG peptide (Sigma - F4799) prepared in flag-to-HIS buffer without Triton X-100. The combined eluates were incubated with His-tag Dynalbeads (10103D Invitrogen-ThermoFisher, Waltham, Ma, USA) for 0.5 h at RT with rotation. The His-tag beads were washed twice with flag-to-His buffer without triton X-100 and three times with 50mM ammonium bicarbonate buffer. After the last wash all the buffer was removed, and the beads were flash frozen in liquid nitrogen and stored at -80°C until submission for trypsin digest and mass spectrometry. Prior to submission, small samples from different purification steps were run on polyacrylamide gels (BioRad Laboratories, Inc., Hercules, Ca., USA) which were then rinsed in double distilled water and silver stained using a BioRad silver stain plus kit, according to manufacturer's instructions. Protein samples were reduced and alkylated using 10 mM TCEP in 50 mM ammonium bicarbonate for 1 h at 37 °C and 20 mM iodoacetamide for 30 min in the dark at RT, respectively. 1 µg of trypsin was added to the beads and samples were incubated at 37 °C overnight. The samples were then acidified and supernatants dried down in new tubes. The tryptic peptides were dissolved in 5% ACN/0.1% formic acid and 5 µL was injected to an LTQ-Orbitrap Velos Pro (ThermoFisher Scientific, MA) coupled with a U3000 RSLCnano HPLC (ThermoFisher Scientific). The liquid chromatography and mass spectrometer settings followed those previously described [9].

***Data Analysis****.*

Scaffold (Proteome Software Inc., Portland, OR; v.4.8.9) was used to validate MS/MS based peptide and protein identifications. The UniProt Database was used in determining peptide and protein identification which were accepted if they could be established at greater than 99.0% probability. The Scaffold Local FDR was used and only peptides probabilities with FDR 0.1% were used for further analysis. The Normalized Total Spectral was selected for the quantitative measurement estimate of the protein abundance of individual proteins in samples.

***REFERENCE***

1. Mertens I, Vandingenen A, Johnson EC, Shafer OT, Li W, Trigg JS, De Loof A, Schoofs L, Taghert PH. PDF receptor signaling in *Drosophila* contributes to both circadian and geotactic behaviors. Neuron. 2005; 48:213-9. PMID: 16242402.
2. Klose M, Duvall L, Li W, Liang X, Ren C, Steinbach JH, Taghert PH. Functional PDF Signaling in the *Drosophila* Circadian Neural Circuit Is Gated by Ral A-Dependent Modulation. Neuron. 2016; 90:781-794. PMID: 27161526.
3. Im SH, Taghert PH PDF receptor expression reveals direct interactions between circadian oscillators in *Drosophila.*  J Comp Neurol. 2010; 518:1925-45. PMID**: 20394051**.
4. Liang X, Holy TE, Taghert PH. A Series of Suppressive Signals within the *Drosophila* Circadian Neural Circuit Generates Sequential Daily Outputs. Neuron. 2017; 94:1173-1189. PMID: 28552314.
5. Lam VM, Beerepoot P, Angers S, Salahpour A. A novel assay for measurement of membrane-protein surface expression using a β-lactamase. Traffic. 2013 14(7):778-84. PMID: 23574269.
6. Beerepoot P, Lam VM, Salahpour A. Measurement of G protein-coupled receptor surface expression. J Recept Signal Transduct Res. 2013; 33(3):162-5. PMID: 23557016.

7. Roland AB, Ricobaraza A, Carrel D, Jordan BM, Rico F, Simon A, Humbert-Claude M, Ferrier J, McFadden MH, Scheuring S, Lenkei Z. Cannabinoid-induced actomyosin contractility shapes neuronal morphology and growth. Elife. 2014; Sep 15;3:e03159. PMID: 25225054.

8. Tian X, Zhu M, Li L, Wu C. Identifying protein-protein interaction in Drosophila adult heads by Tandem Affinity Purification (TAP). J Vis Exp. 2013; 5;(82):50968. PMID: 24335807.

9. Huang H, Alvarez S, Bindbeutel R, Shen Z, Naldrett MJ, Evans BS, Briggs SP, Hicks LM, Kay SA, Nusinow DA. Identification of Evening Complex Associated Proteins in Arabidopsis by Affinity Purification and Mass Spectrometry. Mol Cell Proteomics. 2016; 15(1):201-17. PMID: 26545401;
